# Micro/Nanosystems for Magnetic Targeted Delivery of Bioagents

**DOI:** 10.3390/pharmaceutics14061132

**Published:** 2022-05-26

**Authors:** Francesca Garello, Yulia Svenskaya, Bogdan Parakhonskiy, Miriam Filippi

**Affiliations:** 1 Molecular and Preclinical Imaging Centers, Department of Molecular Biotechnology and Health Sciences, University of Torino, Via Nizza 52, 10126 Torino, Italy; francesca.garello@unito.it; 2 Science Medical Center, Saratov State University, 410012 Saratov, Russia; yulia_svenskaya@mail.ru; 3 Faculty of Bioscience Engineering, Ghent University, Coupure Links 653, B-9000 Ghent, Belgium; bogdan.parakhonskiy@ugent.be; 4 Soft Robotics Laboratory, Department of Mechanical and Process Engineering, ETH Zurich, 8092 Zurich, Switzerland

**Keywords:** magnetic targeting, micro-systems, nano-systems, drug delivery, nanoparticles, microparticles, targeted delivery, magnetic guidance, theranostics, imaging

## Abstract

Targeted delivery of pharmaceuticals is promising for efficient disease treatment and reduction in adverse effects. Nano or microstructured magnetic materials with strong magnetic momentum can be noninvasively controlled via magnetic forces within living beings. These magnetic carriers open perspectives in controlling the delivery of different types of bioagents in humans, including small molecules, nucleic acids, and cells. In the present review, we describe different types of magnetic carriers that can serve as drug delivery platforms, and we show different ways to apply them to magnetic targeted delivery of bioagents. We discuss the magnetic guidance of nano/microsystems or labeled cells upon injection into the systemic circulation or in the tissue; we then highlight emergent applications in tissue engineering, and finally, we show how magnetic targeting can integrate with imaging technologies that serve to assist drug delivery.

## 1. Introduction

Over the last decades, therapeutics have been developed in the form of proteins, peptides, nucleic acids, small molecules, and even cells [1,2,3,4]. Designing effective strategies for targeted delivery of these bio-active agents (bioagents) has emerged as a cornerstone for future pharmaceutical research [5]. Large efforts in drug development are currently dedicated to engineering systems that can spatiotemporally control drug activity and address specific therapeutic needs [3,4,6,7,8,9]. In particular, drug carriers have to overcome biological barriers at systemic, microenvironmental, and cellular levels that are heterogeneous across patient populations and diseases. Micro and nano-sized delivery systems have largely proved to be advantageous in controlled drug delivery [5,10,11,12], and are optimized in increasingly specified ways and a more personalized fashion, confirming that controlling drug dosing and targeting has become essential in the era of precision medicine [5].

Among the multitude of micro/nanocarriers developed thus far, those endowed with magnetic responsiveness stand out from the crowd as they display remarkable abilities stemming from their magnetic nature. These magnetic carriers (MCs) can be formulated by enriching or combining conventional constituents of micro/nanocarriers (lipids, proteins, polymers, or inorganic materials) with magnetically active components with ferromagnetic, paramagnetic, or superparamagnetic nature. For example, one magnetic component that is typically used is iron oxide, extensively applied in the form of particulate material, like iron oxide nanoparticles (IONPs). 

The resulting MCs are popularly customized for disease detection and treatment, as they can be loaded with large amounts of drugs, nucleic acids, or imaging contrast agents [13], and chemically modified to present targeting vectors to localize into the target sites with high precision. In addition, under the application of external magnetic fields, they can be physically displaced allowing one to remotely control their position and movements. Moreover, MCs can locally release energy in the form of heat that can destruct the cells in the surroundings, a strategy known as “hyperthermic therapy” that is widely applied in cancer treatment. Interestingly, in recent years, it has been also demonstrated that, when exposed to external magnetic fields, MCs can apply localized mechanical stimuli on cells and control their behavior by affecting the molecular signaling involved in various cellular processes, such as proliferation, adhesion, and differentiation. Finally, MCs can be visualized via in vivo imaging techniques. MCs can serve as contrast agents in magnetic resonance imaging (MRI), but as they can be functionalized with diverse imaging tags, they can be also visualized by other imaging techniques. Therefore, the magnetic nature of the MCs offers the unique chance to remotely control the localization and therapeutic action of bioagents, while visually monitoring the targeted delivery process. In addition, the micro and nano-capacities of these systems make it possible to load therapeutics of various types, ranging from small molecules to nucleic acids and cells. Sensitive bioagents can be protected by encapsulation within internal compartments of larger systems, like microcapsules. Nanosized MCs, like nanoparticles, can be surrounded with surface coatings that provide accessible sites for conjugation with functional molecules. 

Such versatility has promoted the use of MCs in a plethora of advanced biomedical applications that include genetic engineering, gene therapy, cell therapy, microrobotics, and tissue engineering. For these applications, MCs have been engineered as multifunctional platforms that combine mechanisms of controlled drug release, activatable cytotoxicity, spatial guidance, and multimodal imaging. In particular, the magnetic guidance can minimize drug dissemination to unspecific tissues, and can therefore substantially improve the bioagent efficiency and decrease the required dosage. 

Even though the potential benefits of MCs are remarkable, certain critical aspects have to be considered in view of their full integration into the biomedical practice. For example, the constituent materials that impart magnetic properties to these systems are typically associated with biocompatibility issues. Moreover, the magnetic responsiveness and controllability of MCs depend not only on the nature of the magnetic component but also on the design parameters (such as the carrier geometry and size). The understanding and prediction of the magnetic behavior of MCs, therefore, requires the simultaneous assessment of their composition, molecular structure, and overall morphology. The magnetic and morphological characterization must then be put into the context of using MCs within biological environments. Only this way, one can identify those parameters that affect the magnetic properties and potentially alter the efficacy of the MCs in biomedical applications. 

**Figure 1:**
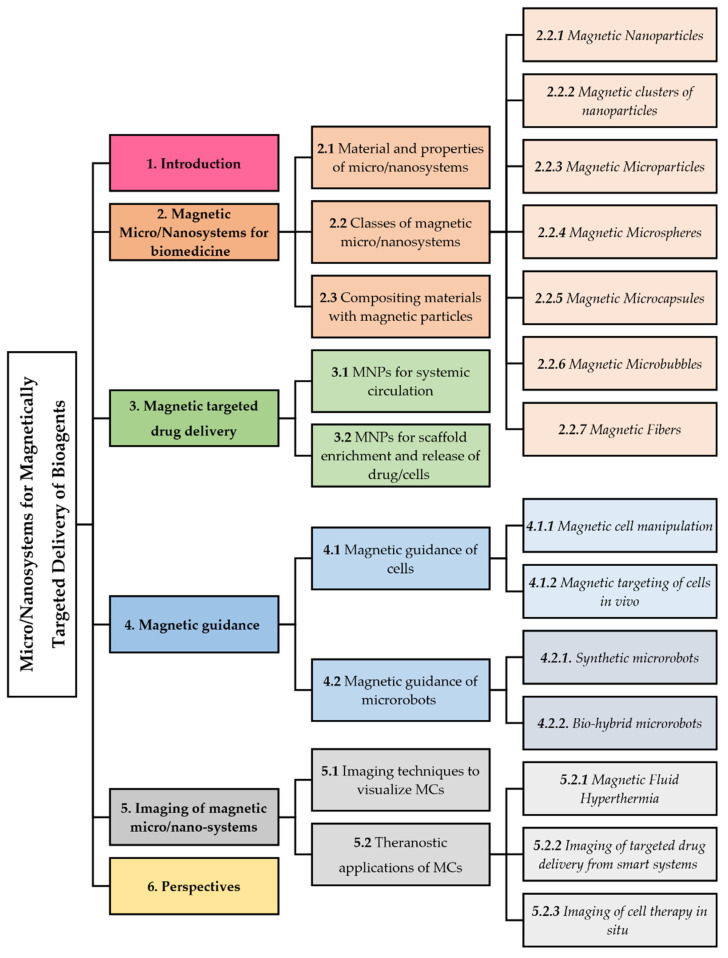
**Schematic diagram of the review contents.**

MCs can become the next generation of multimodal systems for advanced magnetically targeted delivery as they can act under the remote, high penetration, and non-invasive guidance mediated by magnetic forces. However, to fully realize the MCs’ potential, their movement, affinity to the target sites, and cargo release ability have to be assessed while taking into account the specific magnetic behavior of the carrier. The design of magnetic devices has been extensively reviewed by Liu et al., and will not be discussed here [14]. In this review, we will discuss the utility of MCs in the magnetically-aided targeted delivery of various bioagents (Figure 1). We will first introduce the most widely used categories of MCs, and briefly illustrate their properties and association with diverse types of bioagents. Second, we will provide an overview of the application of MCs to drug delivery, highlighting those advantageous features that have caused the recent breakthrough of MCs in this field. Furthermore, we will describe the combination of MCs with external magnetic forces for the spatial guidance of cells and microrobotic systems, and discuss the imaging trackability of MCs within the context of drug delivery. Finally, we will critically point out the core challenges that have to be addressed to make these systems effective in specific medical applications and translatable to human use and propose future directions in advancing control delivery of pharmaceutics via MCs. 

## 2. Magnetic Micro/Nanosystems for Biomedicine 

**Figure 2:**
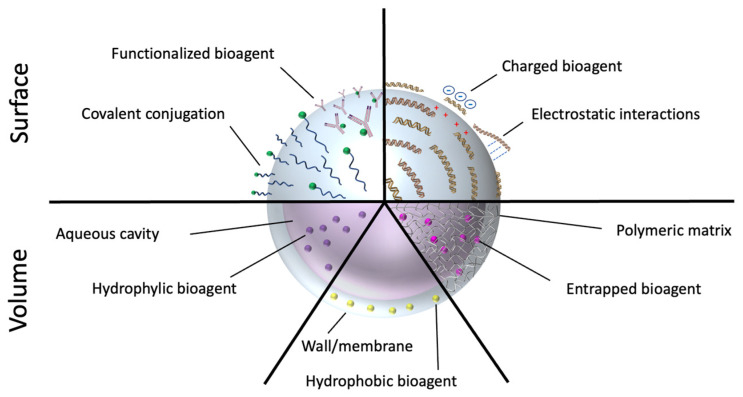
**Drug loading on drug delivery platforms.** Drugs can be loaded onto nano- and microcarriers via attachment to the surface or incorporated into the whole carrier volume. Bioagents can be covalently or non-covalently conjugated to the surface, or entrapped into the internal cavities or the constituent materials of the particles.

Micro/nano-carriers can store large payloads of drugs, and protect them during vehiculation within physiological systems [11,15,16]. These platforms can have a myriad of different morphologies, in which drugs can be physically entrapped in the matrix, encapsulated in inner cavities, or attached to the platform’s structure via different mechanisms of adhesion. Hollow architectures offer large inner compartments where bioagents can be stored. These systems include, for instance, microspheres or nanovesicles made of lipids, dendrimers [17,18,19,20], polymers [21], proteins [22,23], natural membranes, or other structures of biological origin (e.g., exosomes, extracellular vesicles, or yeast and bacterial capsules) [24,25,26,27]. Solid particles typically consist of inorganic materials, polymers, or proteins, and can entrap drugs within the matrix or carry them as attached to the surface (Figure 2).

**Figure 3:**
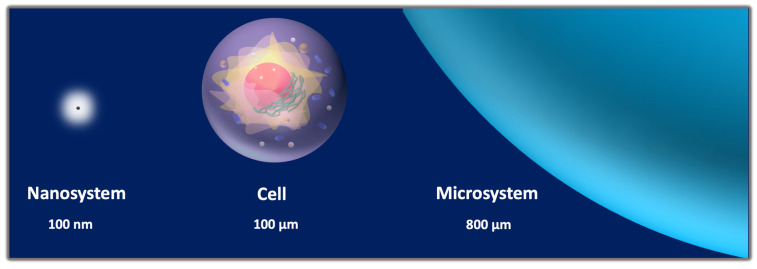
**Nano to micro-scale.** Comparison among the typical size of nanosystems, microsystems, and cells: nanoparticles have diameters comprised between 1 and 100 nm; microparticles have diameters ranging from 1 µm to 1000 µm, whereas the diameter of cells can vary from 10 to 100 µm.

Albeit with some differences depending on the shape and composition of the carriers, their nano- or micro-size guarantees an optimal interaction with various biological entities present in the biosystems, which range from small molecules to macromolecules, organelles, and cells (Figure 3) [28]. Nevertheless, the size difference between nano- and microparticles proffers several effects on the drug loading, permeability across the biological membranes, intracellular processing, and kinetics of physiological distribution and tissue retention [29]. On the one hand, being much smaller than human cells, nanosystems can penetrate the mucosa, safely circulate into the bloodstream, and interact with cellular and subcellular targets. As such, nanomaterials can be applied to intracellular or intranuclear drug delivery, as well as to modulation of biochemical cell pathways by interacting with membrane molecules. On the other hand, microparticles could be most useful as reservoir systems for controlled drug release and protection of bioagents from enzymatic degradation. 

Nano/micro drug carriers can be provided through all administration routes, however, there are some limitations for the systemic administration of microcarriers that depend on the size. When drug carriers are released into the bloodstream, they move in the body following kinetics and distribution patterns that mostly depend on the specific physicochemical properties of the carrier (passive targeting) [30]. However, nano/micro-platforms can also carry and accurately deliver their content to desired destinations (active targeting). Targeted carriers can be produced by molecular functionalization of the nano/micro-systems with targeting vectors that home preferentially into target tissues [10,15,30]. Such targeting moieties provide the systems with the ability to localize into precise areas of the body, leading to improved therapeutic efficacy and reduction in off-target toxicity [3,31]. Stimuli-responsive or biodegradable materials can be used to exert spatiotemporal control over the delivery of the bioactive agents [9,32,33,34,35]. Nevertheless, these systems have be to designed through criteria that depend on the unique structure of the target tissue and organs [4].

The functionality of micro/nano-carriers can be augmented with remote controllability by imparting magnetic responsiveness to the platform structure. Carriers can be magnetized by using magnetic materials as structural bulk components or additive constituents. Magnetic targeting is based on the general assumption that MCs can be spatially controlled within living beings via magnetic fields. In particular, when the MCs are intravenously/endovascularly administered, they can accumulate within the area to which the magnetic field is applied. In addition, the local magnetic agglomeration can provide detectable imaging signals to track the local drug delivery. Structural features of the carriers, physiological parameters of the environment, and technical parameters of the experimental setting determine how effectively the MCs accumulate. Particle size, surface characteristics, as well as field strength, and features of the circulation system (like the blood flow rate) can influence the distribution of MCs at various extents. Local magnetic fields generated by permanent magnets are applied to facilitate the extravasation of carriers into the targeted area.

In the following sections, the most commonly used in MC design magnetic materials will be identified, and the classes and properties of the resulting carriers will be described. Innovative and more conventional applications of MCs will be exemplified to show how the versatility of MCs spans various domains of biomedical research and practice. Finally, the utility of MCs in targeted delivery will be discussed in view of their biophysical and magnetic properties.

### 2.1. Material and Properties of Micro/Nanosystems

Biomedical MCs can be obtained by incorporating a magnetically active phase in the structure of the delivery platform. As a key aspect, MCs display a magnetic fingerprint with peculiar features, such as saturation magnetization, magnetic anisotropy, and magnetic behavior in the presence of applied magnetic fields [36,37,38,39]. These features greatly vary according to the particle size and magnetic structure that can account for a single- or multi-domains organization [37,40,41,42,43]. For a better understanding of magnetism in micro and nanosystems, the reader is referred to extensive, specialized reviews on the topic [38,44]. In the following paragraphs, we will briefly survey the composition of MCs and their relevant properties for biomedical applications. 

Among magnetizing materials, the magnetic spinel ferrites M_x_Fe_3−x_O_4_ (M = Mg, Mn, Fe, Co, Ni, Cu, Zn) have been extensively used to produce drug delivery systems that could overcome the major limitations found in nonmagnetic carriers, namely the poor site-specific localization and the high recognition and clearance by the effector cells of the reticuloendothelial system. Various preparations of microspheres with porous or hollow/porous architectures, as well as solid nano-sized particles were formulated from ferrites. In fact, the high magnetism of ferrites imparts high magnetic responsiveness to carriers, so that these compounds can create micro/nano-objects that can be controlled during navigation within blood vessels. Ferrite-based carriers can be irradiated with microwaves and produce heat that triggers the release of the loaded drugs [45]. Nevertheless, iron oxide, typically formulated as IONPs, has been the most extensively involved in the production of biomedical micro and nanosystems. Magnetite (Fe_3_O_4_) and maghemite (γ-Fe_2_O_3_) are biocompatible forms of iron oxide that have been intensively exploited to produce biomedical IONPs. These nanoparticulate materials are composed of a magnetic core further surrounded by external coatings that cover the surface of the nanoparticle to increase its biocompatibility or provide suitable sites for functionalization. Coatings typically consist of inorganic materials, phospholipids, or biocompatible polymers. For example, the dextran-based coating has been employed in a variety of MCs and many of these formulations have reached advanced clinical trials or are already marketed. The magnetic core has paramagnetic or superparamagnetic properties, which render these objects visible by MRI. The popularity of IONPs derives highly tunable properties (such as size and surface characteristics) that simplify the engineering of systems customized for specific applications or endowed with smart behavior, and multifunctionality. Moreover, iron oxide is generally regarded as a biocompatible material suitable for cell experiments and safe use in living beings. In fact, iron is an essential element in human or animal bodies. As such, within relatively wide ranges of dosage and exposure, iron oxide can be efficiently processed through the physiological biochemical activities of the cells and enter the healthy metabolism of organisms. The stability of IONPs varies depending on the specific features of the system (e.g., the robustness of the coating) and the conditions of the biological environment in which the nanosystem is used. However, in general, IONPs are considered stable particles that can remain inside the cells for long time ranges (even months). As a consequence, after being administered to cells and living beings, IONPs can be safely decomposed, but typically through slow degradation kinetics. In recent years, experimental evidence has raised novel doubts in regards to toxicity. Cellular damage was observed in response to contact with IONPs and attributed to the generation of reactive species of oxygen (ROS).

### 2.2. Classes of Magnetic Micro/Nanosystems

**Figure 4:**
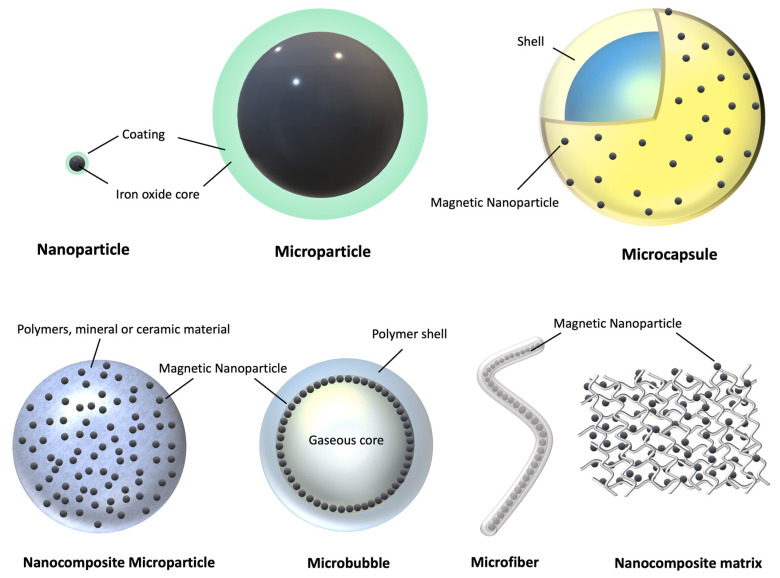
**Different morphologies of micro/nanosystems for the magnetic targeted delivery of bioagents.** Magnetic carriers can be manufactured to have different structural characteristics and classified according to their size and morphological features into: nanoparticles, microparticles, microcapsules, nanocomposite particles and matrices, microbubbles, and microfibers.

The magnetic systems, which are being explored for magnetic targeting, can be categorized into various classes according to their size and morphology. In regards to drug loading/release and magnetic manipulation, the most promising classes of MCs are: nanoparticles, microspheres, microcapsules, microbubbles, fibers, nanoparticle clusters, and nanocomposite matrices (Figure 4). The following paragraphs will survey the properties of MC classes and how these characteristics can be exploited for the optimal use of MCs as bioprobes in therapeutic delivery. 

#### 2.2.1. Magnetic Nanoparticles

Magnetic nanoparticles (MNPs) are magnetic materials on the nanometer scale that have remarkable properties that evoked enormous interest in life sciences in the last decade. In particular, IONPs have emerged as diagnostic and therapeutic tools as their fundamental properties as nanomagnets, defined at the nanoscale, can be extremely useful in biological applications. IONPs are composed of magnetite or its oxidized form maghemite and have diameters ranging between about 1 and 100 nm. According to their physical properties, IONPs show different magnetic natures. Ferromagnetic particles are characterized by a permanent mean magnetic moment [46]. This comes together with a large effective magnetic anisotropy, which suppresses the thermally activated motion of the magnetic moments within the particle core. These features distinguish the ferromagnetic particles from the superparamagnetic ones. Superparamagnetism is found in MNPs with particle size smaller than a certain critical size (typically particle diameter less than 30 nm in IONPs). Below this size limit, IONPs are single-domain; namely, they are composed of one magnetic domain only. In such a condition, the overall magnetization of the MNP acts as a single giant magnetic moment that derives from summing the totality of individual magnetic moments carried by the MNP atoms. This assumption is referred to as “macro-spin approximation”. The magnetization of the superparamagnetic IONPs (SPIONs) randomly flips direction under the influence of temperature, but it can also be aligned using an external magnet. In the absence of an external magnetic field, the measured magnetization of superparamagnetic nanoparticles is null (superparamagnetic state). External magnetic fields can magnetize the nanoparticles similarly to a paramagnet, but with a larger magnetic susceptibility. Once the external magnetic field is removed, the SPIONs lose their magnetization. The explanation is that, in superparamagnetism, thermal excitation causes the dipole moment of a single-domain particle to fluctuate so rapidly that no magnetic moment for macroscopic time scales can be observed. As such, superparamagnetic particles are non-magnetic in the absence of external magnetic fields but do develop a mean magnetic moment when magnetic fields are applied. 

Superparamagnetism is one of the most attractive properties in the use of MNPs. In fact, the reversible magnetization ability of SPIONs has proven to be very useful to guide and manipulate them from distance through magnetic fields. This ability was transferred to cell applications, making it feasible to manipulate cells that have associated with MNPs or have internalized them. As such, SPIONs have been used for: labeling cells; separating them from complex samples or preparations; spatially guiding them; conditioning the release of drugs; remotely controlling actuatable microsystems (microactuators). 

Whereas MNPs manufactured from other highly magnetic materials (like Co and Ni) are limited in their biological applicability by issues of toxicity and easy oxidization, the IONPs have instead found extensive application in gene and drug delivery, bioimaging (MRI and magnetic particle imaging), and magnetic ferrofluid hyperthermia, thus emerging in medical diagnosis and therapy. Moreover, IONPs are also used in the production of terabit magnetic storage devices and sensors, as well as for catalysis, separation of cells and biomolecules (protein separation, DNA/RNA fishing), and superparamagnetic relaxometry. 

IONPs can be produced via different wet chemical, microbiological, and dry processes that can be classified as chemical, biological and physical methods [47,48]. Each technique provides manufacturers with different abilities to tailor the geometry, stability, crystallinity, dispersion trends, and magnetic properties of the nanoparticles [41]. Chemical synthesis can occur in many ways in techniques such as the chemical coprecipitation, electrochemical method, sol-gel method, flow injection method, supercritical fluid method, hydrothermal reaction, thermal and sonochemical decomposition, and nanoreactor techniques [43,47,49]. Whereas biological synthesis is based on the activity of fungi, bacteria, plants, and proteins [50,51], physical synthetic methods include: pulsed laser ablation, laser-induced pyrolysis, aerosol, power ball milling, gas-phase deposition, and electron beam lithography [47]. Physical methods are often complex procedures that guarantee only limited control of the size of particles in the nanometer range [52]. Although ensuring low cost, reproducibility, scalability, and high yield, the biological methods are time-consuming [51,53,54]. For these reasons, chemical synthesis has been the most widely employed approach as it provides a simple and reproducible route to synthesize IONPs with controlled size, composition, and morphology [55,56]. For example, in the co-precipitation method, IONPs are generated from the co-precipitation of Fe^2+^ and Fe^3+^ by the addition of a base, a process that can be controlled by varying the type of salt used, or adjusting the conditions such as the Fe^3+/2+^ ions ratio, pH, and ionic strength [41].

Coating the IONPs primarily aims at protecting the core from oxidation, so that magnetism can be maintained for longer times. In addition, the nanoparticulate is also protected from aggregation, biodegradation, structural alteration, or co-assembly with components of biological systems. SPIONs are typically coated with biocompatible polymers of synthetic or natural origin, that include dextran, starch, polyethylene glycol (PEG), polyvinyl alcohol (PVA), and poly(L-lysine) (PLL) among others. These polymers offer chemical binding sites to attach drugs or receptors/ligands enabling them to recognize molecules exposed on the membrane of specific cell types. IONP surface functionalization through organic and inorganic corona shells, grafted ligands, and selective-site moieties facilitates the bonding of the nanomagnets to natural biomolecules [48]. Moreover, certain coatings enable avoid accelerated blood clearance of injectable nanomaterials and extend their circulation times, resulting in improved performances in both disease imaging and treatment. In fact, coatings can be made of some polymers escaping from the identification by effectors of the immune system. Alternatively, biomimetic delivery systems are also available, that exploit the cell membrane-based coating technology. 

Even though SPIONs are generally regarded as safe biomaterials, in the past many formulations approved for clinical use were withdrawn shortly after approval [57,58,59,60]. In fact, it has to be mentioned that the cellular response to the exposure to these particles can modulate cell behavior in complex ways, and an understanding of the underlying mechanisms is still required from a generic point of view, namely about the bioeffects that are dependent on common constituents (iron oxide). In addition, the specific features of the various formulations also have to be carefully considered, as the size and surface functionalization of SPIONs can strikingly affect the toxicity. Therefore, studies focusing on the biological distribution and metabolic pathways of the MNPs in cells or bodies will lead the future evolution of research in this field [61].

#### 2.2.2. Magnetic Clusters of Nanoparticles 

Since the poor magnetization shown by individual SPIONs is unfavorable to dragging them through the body, clustering strategies were proposed to enhance the magnetic responsiveness. When combined with other materials, MNPs can be grouped to form nanometric assemblies with higher magnetic controllability and responsiveness. For instance, multilayered magnetovesicles carrying cargos in the hollow cavities can be prepared by self-assembly of IONPs grafted with poly(styrene)-b-poly(acrylic acid) block copolymers [62]. In such platforms, IONPs can be packed at a high density, substantially increasing their magnetization and the transverse relaxivity rate, an essential property that induces MRI to signal variations that eventually result in generating negative contrast. Moreover, magnetovesicles can efficiently accumulate in tumors by magnetic targeting. Clusters of magnetite nanoparticles were developed by Wang et al. to perform synergic photothermal and chemotherapy with controlled localization and drug delivery abilities [63]. The accumulation of these bioprobes at the tumor site was enhanced by directing them through an external magnetic field. Intriguingly, the clusters, which were intended for mitochondrial targeting, were enclosed into a double shell system. Briefly, an inner coating made of polydopamine functionalized with triphenylphosphonium (TPP) was conjugated to an outer shell of methoxy PEG through disulfide bonds. When entering cells, these clusters lost their external shell due to a redox reaction that exposed the TPP sites to mitochondrial recognition. Polydopamine in the inner shell acted as a photosensitizer, causing the mitochondrial membrane potential to decrease. The simultaneous release of doxorubicin (DOX), an anticancer drug, damaged the mitochondrial DNA inducing cell death.

The aggregating materials within the MNP-clusters can have specific properties that impart additional functionalities to the clusters. One example is represented by the multifunctional micelles developed by Zheng et al. This platform was composed of hyaluronic acid-C_16_ copolymers synthesized via peptide formation process. After synthesis, SPIONs and therapeutic agent docetaxel (DTX) were co-encapsulated. The resulting systems were highly biocompatible and biodegradable clusters capable of binding to the CD-44 receptor (overexpressed in various cancer types) through the hyaluronic acid and promoting the uptake via receptor-mediated endocytosis. In another work, IONPs and polydopamine were combined to realize platforms for combined diagnostics and therapy (theranostics) of tumors [64]. These clusters displayed an enhanced magnetic response for the better delivery of anticancer drugs via magnetic targeting. In addition, polydopamine on the surface enabled near-infrared (NIR) photothermal tumor treatment. 

#### 2.2.3. Magnetic Microparticles 

Microparticles are defined as particles with a diameter of 1–1000 μm, irrespective of their architecture. Such a term does not identify any precise interior or exterior structure, while more information in this regard is provided by microparticles subcategorization. For instance, the term “microspheres” precisely indicates microparticles with spherical morphology, whereas “microcapsules” are defined as micro-objects comprising of a core that is surrounded by a material that is distinctly different from core constituents [65]. The following paragraphs survey the main classes of magnetic microparticles that include microspheres, microcapsules, microbubbles, and microfibers. 

#### 2.2.4. Magnetic Microspheres

Microspheres are spherical particles with diameters ranging from 1 to 1000 μm [29,66,67] that have been used for decades in the field of drug delivery in vivo [68,69,70,71]. Biomedical microspheres have been traditionally used to load therapeutic substances and deliver them to the target sites. In time, they have evoked increasing interest in releasing drugs in a sustained and controlled fashion. For such a purpose, they can be prepared from biodegradable proteins or synthetic polymers with tunable chemical properties and controllable deterioration rates [72,73,74]. Drug-incorporating microspheres can be generated via different approaches based on: emulsion solvent evaporation; emulsion cross-linking; coacervation method; spray drying technique; emulsion-solvent diffusion; multiple emulsions; ionic gelation; and self-assembly techniques including layer-by-layer formation [75].

Microspheres can be easily administered through a syringe needle, and carry several pharmaceutical types, like drugs, vaccines, antibiotics, and hormones. In addition, microspheres have an important additional feature: they can entrap and protect cells, therefore serving also as a platform for cell applications in vitro and in vivo. The components of the microspheres can mimic the 3D matrix found in the native cell environment. Moreover, their high surface area-to-volume ratio increases the cell loading ability. Microspheres are well-characterized systems, and many of their properties can be easily modeled. Diffusion and mass transfer behavior of the encapsulated molecules can be understood and predicted to define the precise kinetics to control the drug escape and entry into the body fluid. Various commercial microsphere-based formulations are already available (including Lupron Depot^®^ and Nutropin Depot^®^). 

Micron- and submicron-sized polystyrene [76] and porous mineral [77] spheres can effectively penetrate hair follicles due to the “geared pump” effect [78] and deliver the payload to the deep layers of skin and the blood vessels supplying these layers [77]. In addition to biodegradable microspheres for sustained drug release, also bio-adhesive microspheres have been proposed. These systems can adhere to the mucosal membranes (such as buccal, ocular, rectal, nasal, etc.) and transfer drugs during long residence times. Another example is represented by the floating microspheres that remain buoyant in the stomach, as their bulk density is lower than the one of the gastric fluids. From these systems, the drug release occurs slowly at the desired rate, minimizing the risk of striking and dose dumping. More recently, activatable microspheres that respond to external stimulation have offered smart strategies to achieve localized therapeutic responses in specific organs, improved drug utilization and bioavailability, reduced adverse effects, and prolonged release in situ. In this context, magnetic microspheres with a ferromagnetic nature have emerged for various reasons. In general, magnetic forces and materials are well tolerated by living beings, and magnetic fields have high penetration depth. The factors render the magnetic control safe and effective in whole-body applications of drug release. Moreover, magnetic microspheres can be synthesized as small enough to circulate through capillaries without causing embolic occlusion, namely with size <4 μm. These microspheres retain sufficient magnetic susceptibility to be controlled by external magnetic forces while circulating in the bloodstream. Through magnetic fields with flux densities in the range of 0.5–0.8 T, microspheres can be captured within micro-vessels and dragged into the adjacent tissues. However, it has to be considered that, as magnetic microspheres typically contain magnetite, a large fraction of this substance can remain deposited for long times or permanently in tissues. For example, Genina et al. delivered MNP-loaded CaCO_3_ microcarriers in vivo using fractional laser microablation (FLMA). Even if a complete degradation of the microcarriers was observed, seven days after FLMA the released MNPs were still present in dermis [79].

#### 2.2.5. Magnetic Microcapsules

Polyelectrolyte capsules are micro- or submicron-sized drug delivery systems manufactured via sequential adsorption of oppositely charged polyelectrolytes (so-called layer-by-layer assembly, LbL) onto liquid droplets [80,81] air microbubbles [82], cells [83,84], or solid templates with subsequent decomposition of this template [85,86], ease of preparation, control over the shell thickness, and flexibility in the choice of constituents granted the application of polyelectrolyte capsules in materials and life sciences [87,88,89]. In microcapsules, the core can be used to load substances to be concentrated, protected, and delivered [90,91]. However, the LbL technique enables the assembling of therapeutics in between layers of polyelectrolytes as well, allowing one to customize the system. Multifunctional vehicles can be created utilizing the ability to vary the components, incorporate different inorganic nanoparticles and modify the shell with various organic molecules and antibodies [92,93]. Microcapsules can be readily internalized by cells and then retained for weeks or months [86]. As microcapsules can be loaded with IONPs either into the shell [94,95,96,97] or the core [98,99,100], they have been proposed as a novel strategy to create high local concentrations of magnetic materials [101]. Micro-encapsulation not only reduces the cytotoxicity of IONPs, but also improves their incorporation into cells, extends their retention, and notably enhances their magnetic properties [102]. NPs are in fact characterized by more rapid washout after cell internalization, while microcapsules can remain inside cells for longer time ranges. The inclusion of MNPs in LbL microcapsules grants a more rapid sedimentation rate for the capsules in turn and improves their contact with cells promoting the delivery of bioactive molecules to cells. For example, Pavlov et al. demonstrated that additional modification of poly-L-arginine/dextran sulfate microcapsules has significantly improved intracellular luciferase enzyme activity (25 fold) and increased transfection efficiency with plasmid DNA (3.4 fold) [103]. Moreover, magnetic LbL-assembled capsules present a strong magnetic response that derives from the coherent effect of thousands of MNPs tied in one entity [29,104]. In such a manner, they represent a unique delivery system allowing for remote navigation or holding with an external magnet not only in vitro [105], but in complex dynamics of in vivo as well. For instance, Voronin et al. prepared magnetic multilayered microcapsules via LbL deposition of poly(allylamine hydrochloride) and poly(sodium 4-styrenesulfonate), and then demonstrated that these systems could be effectively controlled in a blood flow [104]. In fact, the microcapsules were guided and trapped by external magnetic fields in precise locations of mesenteric vessels in rats, and their position could be maintained when the magnetic field was turned off. Such a concept of magnetic targeting utilizing the external magnetic field has been recently proven to selectively deliver biodegradable submicron capsules to the tumor in a mouse breast cancer model [100]. Further improvement of magnetic targeting for LbL-assembled capsules is required to better overcome the physiological barriers that every drug-carrying platform faces after its intravenous injection (e.g., flow and shear forces, aggregation, protein corona adsorption, phagocytosis, and blood clearance) [106]. Endovascular addressing has been proposed to be combined with external magnetic navigation of polyelectrolyte capsules to enhance the effectiveness of their delivery to a specific site [107]. Comparison of intravenous and intra-arterial administration of biodegradable microcapsules containing MNPs in their shell targeted in the hind paw vessels using an external magnet have demonstrated four times higher efficiency of the latter.

Importantly, the high payload capability offered by microcapsules enables applications where stronger dragging forces are needed, such as in spatial control of cells [108,109]. For instance, IONP-loaded polymeric microcapsules have been recently used to magnetize cells assembled into spheroids, which could be then used for magnetic patterning and biofabrication of tissue-engineering constructs [110]. Magnetic microcapsules are therefore valuable tools for magnetic tissue engineering, in which magnetic forces serve as temporary supports to assemble biological structures from individual cells or spheroids.

In addition to the remote navigation property, magnetic microcapsules possess the ability to activate their functionalities (including the payload release) by responding to specific chemical (e.g., enzymes, decreased pH) or physical triggering stimuli (e.g., light, heat, ultrasound, electric or magnetic fields) [92,111,112]. It is well-illustrated, that the functionalization of LbL-assembled capsules with MNPs enables their controlled opening under ultrasound irradiation and magnetic field. In particular, Shchukin et al. demonstrated that Fe_3_O_4_-containing poly(allylamine)/poly(styrene sulfonate) capsules can be disrupted under 20 kHz-ultrasound irradiation even at short (5 s) sonication times [113]. Importantly, the payload liberation from these capsules was induced at an ultrasonic power of 100 W, comparable to those of ultrasonic generators applied in medicine. Carregal-Romero et al. have demonstrated the magnetically triggered cargo release from the polyelectrolyte capsules decorated with iron oxide nanocubes [114]. The authors used an alternated magnetic field (AMF) to locally heat nanoparticles embedded into the polyelectrolyte shell, thus causing its destruction. Besides spherical polymeric capsules, anisotropic LbL-assembled particles of micrometer and nanometer sizes have gained great attention. Kozlovskaya V. and Kharlampieva E. have recently summarized the most interesting results in the field demonstrating that anisotropy in shape results in shape-dependent chemical or physical properties of the vehicle and plays a crucial role in its interaction with various cell types [115]. Obviously, anisotropic LbL constructs are characterized by a more pronounced bio-mimetic behavior, improved deformation, and recovery properties, as well as by a shaped-directed flow, surface adhesion, and motion. These advantages open up broad prospects for their application in biomedicine as artificial micro/nanomotors or intelligent drug carriers.

Microcapsules showed promises for the design of the next generation of smart drug delivery systems with integrated propulsion. Recently, controllable magnetic microcapsules, named iMushbots, were prepared from fragments of a mesoporous mushroom (*Agaricus bisporus*) coated with magnetite NPs. These intelligent and biocompatible vehicles were capable of autonomous motion based on chemotactic processes and were tested for targeted drug delivery [116]. The magnetite not only enabled the remote motion control of the iMushbots, but also had a powering reaction. In fact, iron oxide promoted a reaction of heterogeneous catalytic decomposition of the peroxide fuel powering a pH-induced chemotaxis process. In addition to inherent propulsion, these microcapsules-based machines smartly associated with the loaded cargos, as they were capable of steadily retaining cationic anticancer drugs in environments with alkaline pH and readily release them at low pH values. This conditioned behavior is very promising for the realization of advanced drug micro-formulations, in which the carriers are highly stable during circulation into human blood, while drug release is instead facilitated in sensitive areas characterized by acidic environments (like the cancerous tissue). 

#### 2.2.6. Magnetic Microbubbles 

Microbubbles are clinical contrast agents for ultrasound imaging consisting of microsystems with sizes of ∼1–10 μm. Microbubbles have coatings that can be functionalized with ligands or targeting vectors, to create targeted systems recognizing biomarkers expressed in diseased tissues [117]. Magnetized microbubbles can be produced by incorporating MNPs in microbubbles’ coating. The magnetic material can be loaded on the outside surface of the microbubbles within the coating, or within an oil layer underneath the coating [118,119]. To properly interact with the shell constituents of the microbubbles and to be efficiently loaded, the MNPs have to be coated with different phospholipids or dendrons, which eventually affect the physical properties and stability of the overall microsystems. Then, the incorporation of MNPs into the microbubble shell can be accomplished by sonication [120]. 

Magnetic microbubbles are visible by ultrasonography, but also responsive to physical triggers delivered by both magnetic and acoustic field changes [121]. Optimal use of magnetic microbubbles in targeted drug delivery requires specialized hardware, such as combined systems for magnetic-acoustic stimulation that guarantee improved performance in vitro and in vivo as compared to the use of two separate devices [122]. It was demonstrated that probes combining a magnetic array and ultrasound transducer in a single unit could simultaneously concentrate and activate the microbubbles at the target site. The co-alignment of ultrasound and magnetic fields led to enhanced anticancer drug deposition in gel phantoms and tumor growth inhibition in mice.

The potential of magnetized microbubbles is well highlighted in one recent study showing that drug release in tumors could be finely controlled by optimizing applied magnetic and acoustic fields, while in real-time monitoring the carriers via ultrasonography [123]. By mixing heparinized MNPs with a suspension of protamine-functionalized microbubbles, the authors prepared magnetic microbubbles that evaded pulmonary entrapment but could be selectively gathered in lung tumors and visualized by ultrasound imaging. In addition, under stimulation with focused ultrasound, the microbubbles collapsed and locally released the carried model drug. 

Magnetic liposomes are another useful system for magnetic drug targeting. Liposomes are biocompatible lipid-based vesicles ranging in size from 50 nm to 1 µm, that can carry drugs or other compounds. They share some analogies with microbubbles, like a vesicular architecture that can be differentially functionalized to attach targeting vectors and load therapeutics of different kinds. MNPs can be encapsulated by liposomes. It was shown that liposomes containing γ-Fe_2_O_3_ nanoparticles doped with anethole ditholethione can accumulate in tumor sites when spatiotemporally navigated by an external magnetic field [124]. This nanoscaled delivery platform could intratumorally convert to microsized bubbles based on hydrogen sulfide pro-drug (H_2_S), through a process that could be dynamically monitored by magnetic resonance and ultrasound dual-modal imaging. Importantly, the H_2_S microbubbles were able to ablate the tumor tissue when exposed to higher acoustic intensity. The H_2_S molecules localized at a high concentration during this process could then diffuse as gasotransmitters into the inner tumor regions providing further antitumor functions. 

#### 2.2.7. Magnetic Fibers 

Magnetically responsive drug delivery systems can also be designed with non-spherical particle morphology. One anisotropic design that recently became popular for specific drug delivery applications is the fiber. Magnetic fibers are fabricated by combining polymeric fibers with MNPs. One example is found in the work of Perera et al., where PVA fibers with rough 100–300 nm diameter and filled with MNPs were synthesized via infusion gyration [125]. Controlled release of drugs could be achieved by magnetic actuation. A model drug (acetaminophen) was uploaded via impregnation and rapidly released by applying magnetic fields, with 90% cumulative release achieved in 15 min only during magnetic actuation. 

Drug release on demand from a fiber-shaped delivery system was triggered in response to varying pH and temperatures [126]. In such a case, magnetic bioactive glasses complexed with the antitumor drug Cisplatin were doped into the chitosan-grafted poly(ε-caprolactone) (PCL) nanofibers. Compared with control fibers kept under physiological conditions, the fibers exposed to a pH value of 5.5 and a temperature of 43 °C displayed augmented Cisplatin release rates. Following these observations, MG-63 osteosarcoma cells were treated with simultaneous chemotherapy and hyperthermia by exposure to an alternating magnetic field that activated the magnetic fibers.

Core-shell nanofibers of approximatively 300 nm in diameter were developed by coaxial electrospinning of N-carboxymethyl chitosan-PVA and PCL for the core and the shell, respectively [127]. The antitumor drug DOX and nickel ferrite nanoparticles were incorporated into the nanofibers for extended and controlled release of DOX against MCF-7 breast cancer. DOX release was studied under physiological and acidic pH in the absence or presence of applied magnetic fields, respectively, revealing that the maximum cytotoxicity on cancer cells (83%) could be obtained at day seven by magnetically stimulating nanofibers with chitosan/PCL/nickel ferrite 10 wt.% composition. Another group fabricated a magnetic nanofibrous mat-based bandage for the treatment of skin cancer by non-invasive induction of hyperthermia [128]. PCL fibers incorporating magnetite nanoparticles were obtained by electrospinning and then magnetically stimulated with an external alternating current magnetic field. The fibers locally dissipated heat energy, thus rapidly augmenting the surrounding temperature up to 45 °C. This bandage killed both parental and DOX hydrochloride-resistant HeLa cells, demonstrating enhanced DOX activity caused by increased temperatures. Mice with chemically induced skin tumors fully recovered within one month after five hyperthermic sessions of 15 min.

Due to their peculiar morphology, fibers have attracted considerable interest for drug release within specific environments and applications. Fibers are promising for the treatment of tubular or ductal anatomical structures (e.g., lung airways, blood vessels) or the construction of implantable tissue grafts. For instance, they can be used in controlled pulmonary drug delivery as they may travel deep into the lung airways. In this location, fibers facilitate the accumulation of pharmaceuticals at the target site and enable their sustained release [129]. In this context, electrospun superparamagnetic polymer-based biodegradable microrods were recently proposed. These fibers consisted of a polymeric phase of poly(l-lactide) (PLLA) and polyethylene oxide (PEO), enriched with oleic acid-coated magnetite nanoparticles. Microrods were prepared by exposing the electrospun fibers to ultraviolet (UV) irradiation and sonication, before incorporating methyl 4-hydroxybenzoate as a model drug. The authors showed that the magnetic properties of the microrods were responsible for inducing hyperthermia under AMF. Moreover, airborne microrods could be manipulated by direct current magnetic fields, thus enhancing targeted delivery to lower airways. 

Scaffolds for release on demand of bioagents have been typically generated as porous structures but, in such an architecture the drugs freely diffuse, especially during long-term studies. Having good potential for better drug retention and controllable behavior, activatable magnetic fibers could have preferable morphology and profile to construct scaffolds for tissue engineering or cancer therapies. Wang et al. recently developed scaffolds from hollow fibers composed of alginate/IONPs by coaxial 3D printing [130]. Drugs, proteins, or living cells were encapsulated in the core part of the fibers that was characterized by a lower alginate gel concentration. Magnetic actuation of the fibers resulted in physical deformation of the scaffold under magnetic field exposure and subsequent release of loaded therapeutics. Moreover, due to their elongated structure, the fibers acted as diffusion barriers and minimized the uncontrolled diffusion of bioagents from the non-stimulated scaffolds. Under intermittently magnetic stimulation, a formulation with optimized composition (10 wt.% of alginate, 13% of IONPs) and crosslinking conditions (0.1 M CaCl_2_, 10 s) displayed repeated on-demand release capability in both cell and animal experiments. 

### 2.3. Compositing Materials with Magnetic Particles

Magnetic systems at both nano- and micro-scale can be used to create composite matrices with various applications [131,132,133,134]. Magnetic nano- and micromaterials are typically added to polymeric hydrogels or scaffolds to generate multifunctional materials that combine the properties of the polymers and the magnetic materials. Briefly, to fabricate magneto-polymeric materials, ferri, and/or ferromagnetic particles can be mixed or embedded in a polymeric matrix, and then different strategies can be followed to incorporate the particle phase either transiently or permanently [133,135]. 

Magnetic microcomposites include magnetic microparticles, and as such, they incorporate magnetic materials clustered in microsize sites of accumulation. Incorporating micro MCs, however, imparts a high magnetization to composites that display microscale structural heterogeneities, which might lead to gross alterations of the mechanical behavior and affect their suitability for specific applications. For instance, in additive manufacturing (e.g., deposition-based printing) a careful evaluation of the ink’s mechanical properties is necessary to maintain shape fidelity and printability of the deposited material. Spangeberg et al. realized a magnetic bioink based on alginate and methylcellulose (3 and 9%, respectively) that was incorporated with magnetite microparticles [136]. While shear-thinning properties remain unaltered, viscosity and magnetization of the composite material strongly increased with the magnetic microsystem concentration. Upon optimizing the magnetic microparticle concentration (25% *w*/*w*), scaffolds were fabricated, which could deform under the application of an external magnetic field and represent a promising platform for cell mechanical stimulation and improvement of stem cell differentiation in cell-laden scaffolds.

Due to the high integrability of nanomaterials with macromolecular structures like polymers, nanocomposites display structural homogeneity, monophasic mechanical behavior, and improved physico-chemical properties that let nanocomposites emerge in biomedical applications, such as reinforcement of engineered tissue grafts [131,134,137,138]. In particular, the magnetic responsiveness renders magnetic nanocomposites particularly attractive to realize controllable cell culture models and improve cell functionality within biological tissue artifacts [138]. To include MNPs into polymeric matrices, different strategies can be applied, which include: (*i*) co-precipitation during the matrices synthesis [139,140]; (*ii*) adsorption on pre-formed matrices [141]; (*iii*) adsorption during the freezing process [142]; (*iv*) hydrothermal method [143]; (*v*) sol-gel method [144]. These approaches are based on different technical principles and have their advantages and disadvantages. For instance, the procedure for co-precipitation is a facile and convenient synthetic method, with low environmental impact, as it is carried out in aqueous solution with relatively low reaction temperatures. However, this procedure can affect the synthesis process and requires special conditions, such as high stirring speeds, controlled atmospheres, reaction medium and drying temperatures. On the other hand, the adsorption method is more predictable but limited concerning the loading amount and affected by the physical and chemical properties of the matrices, such as surface chemistry, charges as well as pore size. The recently developed method of freezing incorporation (method iii) is labor-intensive but provides four times higher loading efficiency than the coprecipitation or adsorption method. In simultaneous adsorption-freezing, the repetitive adsorption of the magnetite particles occurs in a water-based solution at a negative temperature. In such conditions, the ice crystallization provides additional pressure onto MNPs, which improves their adsorption efficiency. The hydrothermal method (iv), which uses an autoclave or microwave to reach high pressure and temperature, allows the preparation of a stoichiometric compound with appropriate crystallinity. Although this method is rather specific, the high temperature can affect the matrix stability. In the sol-gel method (v), the gel molecules stick together with the MNPs and the porous matrices. In addition to being fast and efficient, this strategy does not require any specific equipment. However, additional compounds (such as the gel molecules) are present in the final composition, which can influence the surface chemistry properties of the particles.

Besides responding to magnetic fields, the MNPs can enhance the matrix properties, by conferring for instance additional structural stability. For example, adding MNPs to the calcium carbonate particles can stabilize the unstable calcium carbonate in the vaterite phase and delay its recrystallization to calcite from hours to several days [145]. 

## 3. Magnetic Targeted Drug Delivery

Magnetic drug targeting can be realized through different approaches [121]. In the majority of cases, an external magnetic field is applied to control the localization and the functional activity (e.g., drug release) of the MCs [63,104,146,147,148,149]. External magnetic fields can be generated from various kinds of stationary magnets or via electromagnetic coils [121]. For clinical implementation, one has to take into account that, according to the guidelines provided by the International Commission of Non-ionizing Radiation Protection [150], patients could not be subjected to the magnetic flux densities that exceed 400 mT in any part of their body. In clinical practice, one to two T magnets are used to achieve local magnetic field intensities of around 400 mT. In preclinical research, magnetic drug delivery is realized in the presence of applied magnetic fields with intensities varying between 0.1 T and 1.5 T [151,152]. The distance between the magnet and the target site for delivery, as well as the geometry of the magnets, crucially affect the effectiveness of delivery [63,148]. One of the major challenges in magnetic drug delivery is delivering therapeutics within deep tissues in vivo. In particular, one common approach consists of placing stationary magnets on the skin. This way, the MCs can be efficiently guided and controlled within superficial tissue layers, but the target sites below a few centimeters (5 cm) under the skin remain not accessible. In order to drag and focus the MCs into deep tissue, a sequence of actuations can be realized by dynamically controlling magnets according to schemes defined by first-principles magneto-statics and ferrofluid transport models [153]. Such a strategy pushed magnetic nanoparticles to a deep target location. 

Nevertheless, our ability to control the MCs from distance through external magnetic fields remains limited. As an alternative, magnetized implantable scaffolds can be used to attract the MCs into the target organs. In particular, nanodrugs can be delivered in a targeted fashion through implants endowed with high magnetism. Recently, poly(lactic-co-glycolic acid) (PLGA) composited with Fe_3_O_4_ was used to produce durable, biocompatible, and highly magnetic implant scaffolds that can locally attract magnetized chemotherapeutics and destroy cancer cells [154]. Although challenging, such approaches can facilitate more precise treatments and show promise in bone cancer, obesity, and deterioration of the inner ear [155].

### 3.1. MNPs for Systemic Circulation

MNPs are widely designed for the site-specific delivery of therapeutic molecules in the presence of an external magnetic field [156,157]. Chemotherapeutics [158,159], anti-inflammatory drugs [160], immunosuppressants [161], antibiotics [162,163], and antiviral agents [164] can be targeted on the diseased tissue by their use owing to sufficient surface area of MNPs and the possibility to control the payload release with or without a specific trigger. Drug molecules are generally incorporated into the carrying matrix via adsorption and encapsulation approaches or directly linked to the surface of MNPs through the covalent bonding or electrostatic attraction [157,165]. Liberation of the drug then occurs through its leakage from the matrix or as a result of the nanocarrier degradation. This process can be triggered by alternating magnetic fields [166,167] or such stimuli as pH [168], enzymatic destruction [169], and heat [170,171] utilizing linkers that are sensitive to them. Realization of such an approach should guarantee a drastic enhancement of the local drug concentration in the targeted site resulting in therapeutic efficacy increasing. 

Site-specific targeting of the payload is provided either passively based on the vascular bursts [172] and enhanced permeability and retention (EPR) effect of pathologic tissue [173,174] or initiated actively via specific ligand attachment [175,176] or exploiting superparamagnetic properties of the system (magnetic targeting) [14,177]. The latter is an important advantage of MNP-based delivery systems that sets them apart from other nanotechnology-based drugs. The success of such addressing depends on the particle size, surface chemistry, magnetic polarization, and magnetophoretic movability under an external magnetic field [156]. In addition, it requires a good magnet system to guide the drug carriers to the target site [14]. The transport of MNPs in the bloodstream is defined by a dynamic equilibrium between the magnetic and hydrodynamic forces acting on them. Thus, such physical properties as the strength and gradients of the magnetic field, the volumetric and magnetic properties (magnetization) of the carriers, as well as the hydrodynamic parameters (blood flow, hematocrit, viscosity, and the particle concentration in the blood) significantly affects the efficacy of magnetic navigation [151]. 

The possibility of magnetic targeting of drugs has been widely demonstrated utilizing MNP-based carries systemically injected in vivo in different animal models. However, to the moment, no magnetic targeting protocol has successfully passed the regulatory approval to be translated to clinics. Only one MNP-based nanosystem (DOX-loaded MCs) was studied in patients and did not demonstrate convincing results, thus, the study was terminated in phase II/III of clinical development [178]. According to the clinicaltrials.gov database (the website was accessed on 26 February 2022), there is one ongoing clinical trial intended to evaluate the safety, efficacy, and tolerability of intratumorally injected SPIONs accompanied by the spinning magnetic field application in combination with neoadjuvant chemotherapy in osteosarcoma patients (identifying number NCT04316091). However, in this study, MNPs are administered separately from the drug, and their systemic circulation is excluded from consideration. At the same time, the ability to safely and effectively deliver therapeutic moieties to the deep tissue using MNP-based carriers and external magnetic fields remains limited. The main challenges associated with this addressing approach include the design and selection of an appropriate MC considering specific clinical indications, as well as magnet design for deep and precise addressing through the tissue taking into account the anatomical barriers [152]. 

A ligand-receptor-mediated targeting strategy is based on the conjugation of the MNP-based delivery systems with such segments as antibodies, aptamers, endogenous proteins (like, transferrin), hyaluronic acid, folate, and targeting peptides [175,179]. The carrier functionalization with targeting ligands enables their recognition by specific receptors that are overexpressed on the surface of target cells, and thus provides the enhanced accumulation at the desired sites. However, when the modified particles (as well as the non-modified ones) are introduced into a complex biological environment of the circulatory system, the targeting often fails due to rapid clearance by macrophages of the reticuloendothelial system (RES) [180]. This protective mechanism is so potent that the majority of the injected nanocarriers are generally cleared after a few passes through the liver and spleen [181]. 

**Figure 5:**
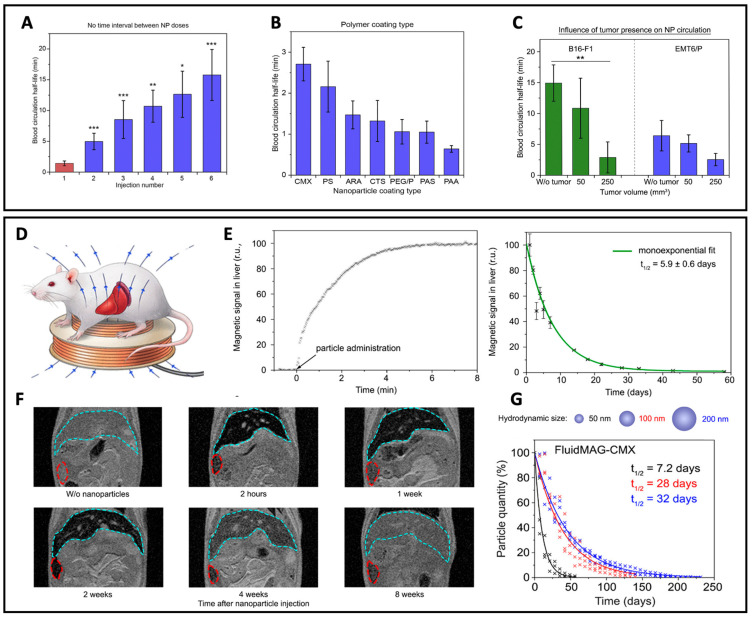
**Circulation and distribution of MCs injected systemically.** MPQ was applied to test the blood circulation and the organ distribution of magnetic particles. (**A**) Influence of repetitive injections of 100-nm MCs on their circulation. Multiple injections of MCs (300 μg) were provided immediately after the clearance of the previous dose. (**B**) Dependence of the half-life time of 100-nm nanoparticles on polymer coating. The data are provided for a dose of 300 μg nanoparticles per mouse. (**C**) Influence of the presence and size of tumors (B16-F1 in C57Bl/6 and EMT6/P in BALB/c mice) on clearance of 50-nm MCs from the bloodstream. The data are provided for a dose of 300 μg nanoparticles per mouse. (**A**–**C**) The experiments were performed on BALB/c mice (blue and red bars) and on C57Bl/6 mice (green bars), n = 3 for each bar. Asterisks indicate significant difference from the control: Welch’s *t*-test, *—*p* < 0.05; **—*p* < 0.01; ***—*p* < 0.001. Adapted with permission from Ref. [182]. (**D**) The degradation of magnetic particles is investigated with the MPQ approach noninvasively in a setup where the magnetic signal from the particles is measured by a magnetic coil placed under the mouse liver and spleen. (**E**) MPQ data of accumulation and degradation kinetics in the liver of 100 μg of citrate-stabilized MPs, and (**F**) representative MRI of the two-month evolution studies in the liver (cyan lines) and spleen (red). (**G**) Dependence of degradation kinetics on particle size for 50, 100, 200 nm MPs coated with carboxymethyldextran. (**D**–**G**). Adapted with permission from Ref. [195]. Copyright 2021 American Chemical Society.

The process of MNPs blood clearance depends on the injection conditions (e.g., particle injection dose and repetition time of administration), nanoparticle properties (e.g., particle size, charge, and coating structure), and animal model (mice strain, absence/presence of tumors, tumor size) was comprehensively studied by Zelepukin et al. (Figure 5) [182]. To register the MCs circulation kinetics, they placed the animal’s tail inside a coil generating a magnetic field on two frequencies and measured the magnetic response of the injected MCs at a combinatorial frequency detecting the particle concentration in tail veins and arteries. This research demonstrated that the injection of low MNP doses (less than 1 mg per mouse meaning 50 μg/g tissue) granted an almost constant half-life time (1–1.6 min), while further dosage enhancement substantially prolonged the particle circulation up to 45 ± 14 min for the 10 mg per mouse dose. This effect was attributed to the saturation of the RES in the organism, which could not eliminate such large nanoparticle doses. Multiple successive administrations of MNPs also resulted in prolonged circulation, starting from the second injection (each additional dose enhanced the effect). At the same time, a one-day pause between the administrations evened out the difference in particle circulation time compared to a single injection. The negatively charged particles demonstrated a slight prolongation of the clearance process. Comparison of the circulation time for 50-, 100- and 250-nm particles coated with glucuronic acid demonstrated a well-established trend: the smaller was the carrier size, the longer its circulation occurred. However, the time difference in clearance between the 50- and 100-nm particles substantially exceeded that between 250- and 100-nm. The effect was again explained by the limited number of receptors on the surface of macrophages that bound nanoparticles, as well as by the overall saturation of the endocytosis process.

Surface modification of the drug-delivering carriers with various coatings (PEG or other hydrophilic polymers) is commonly used to decrease their opsonization and prevent recognition by the immune system, thus inhibiting the non-specific distribution of these carriers [183]. For instance, it was shown that the PEGylated single-core MNPs, which are used in magnetic particle imaging (LS-008 tracer), persisted in the bloodstream of the rats with a half-life of 4.2 ± 0.5 h when injected at the dose of 5 mg of Fe/kg [184]. The PEG-silane coating of SPIONs provided further prolongation of the blood circulation half-life (up to 7 h) [185]. It should be noted that the coating material has a strong effect on blood kinetics depending on the ionic character of the coating material as it influences the particle uptake by macrophages. Obviously, the higher efficiency of particle capture by macrophages is observed, the faster those are cleared from the blood. It was well-demonstrated that phagocytic activity inhibited by decreasing the ionic strength of the coating material deposited on the SPIONs’ surface: the starch, polyacrylic acid, and carboxydextran provided the higher uptake (thus shorter circulation in the blood) of SPIONs than PEG coating [186]. 

Despite the highly valuable advantage of prolonged blood circulation for stealth nanoparticles, the surface coating might bring some negative aspects, like the development of immune response enhancing opsonization for repeated injections (so-called, accelerated blood clearance) [187], inhibiting of the endosomal escape [188] and complication of the cellular penetration of the carriers resulting in a significant loss of delivery system activity [189]. Even though one can guarantee that targeting receptors are not disguised by the protein corona, the coatings can reduce targeting efficiency by hiding the receptor in its own bulky structure [181]. The alternative approaches towards the blood-circulation time enhancement have been proposed so far. Namely, cellular hitchhiking meaning the use of various cells as the particle carriers [190] or the RES blockade (saturation) induced before the MNPs injection [191,192] are exploited to delay the blood clearing and increase targeting capability. Although the suppression of mononuclear phagocyte function is generally achieved by injecting large doses of various nanoparticles [193,194], it can be blocked by the organism’s own intact cells as well. In particular, Nikitin et al. elegantly demonstrated that the pre-induced macrophage saturation with antibody-sensitized erythrocytes (so-called, cytoblockade of mononuclear phagocyte system) increased the circulation half-life of nanoparticles by up to 32-fold [181]. Such cytoblockade was also shown to significantly enhance the therapeutic potential for magnetically-guided drug delivery systems.

The long-term fate of MNPs needs to be addressed when studying their in vivo behavior and effectiveness [156,195]. Comprehensive reviews on their pharmacokinetics, biodistribution, and toxicity have been previously published [196,197]. Briefly, upon the intravenous injection, the MNPs are mostly accumulated within the liver and spleen, which is logical as these organs are responsible for blood purification [156]. The behavior of MNPs in living organisms is significantly influenced by their surface functionalization and physicochemical properties, like the hydrodynamic size, polydispersity index, shape, aspect ratio, stability, and surface charge. In addition, the surface roughness, ζ-potential, and the size of nanoparticles also determine the thickness and composition of the plasma protein corona forming at their surface that, in turn, defines such pharmacokinetic parameters as plasma half-life, distribution, and elimination of the carriers [196,198]. Recently, Zhang et al. have proposed a magnetothermal regulation approach towards the control of protein corona formation on iron oxide nanocarriers in vivo to improve their pharmacokinetics and therapeutic efficacy [199]. They demonstrated that the relative levels of major opsonins and dysopsonins in the corona could be tuned quantitatively by means of heat induction mediated by MNPs under AMF.

Biodegradability of the MNPs is another important issue to be investigated when designing the MNP-based drug carriers aiming at further clinical translation [177], and safe biomedical application of nanoparticles requires a clear understanding of their metabolism [200].

A broad study of the one-year fate of 17 types of MNPs performed by Zelepukin et al. demonstrated a long-lasting effect of the injected dose, hydrodynamic size, ζ-potential, surface coating, and internal architecture of MNPs on their degradation half-life [195]. The authors introduced a magnetic spectral approach for non-invasive in vivo monitoring of MC degradation based on magnetic particle quantification (MPQ) technique (Figure 5). Faster particle degradation was observed for the MNPs of smaller size and negative ζ-potential. In addition, the type and structure of the surface coating were demonstrated again to play a crucial role in their biodegradability.

### 3.2. MNPs for Scaffold Enrichment and Release of Drug/Cells 

**Figure 6:**
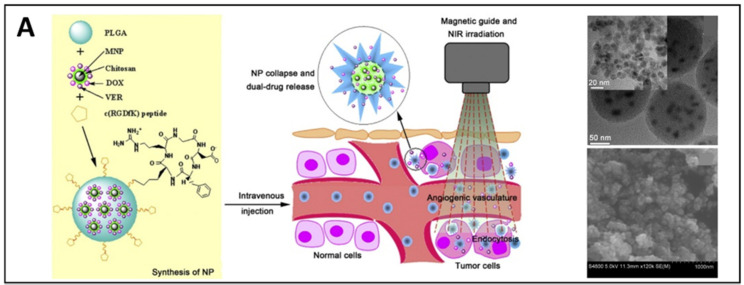

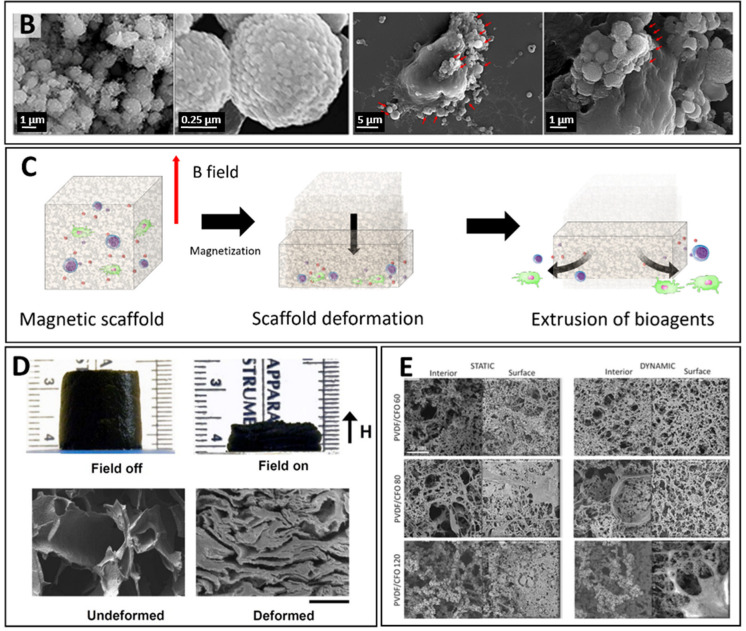
**MCs for drug delivery as injectable agents or magnetizing agents for scaffolds.** (**A**) Drug delivery via injectable MNPs: Shen et al. synthesized MNPs loaded with DOX and verapamil and coated with PLGA. Soon after the injection the mice were treated with a constant magnetic field (about 2000 Oe) laid outside the skin near the tumor site at 25 °C for 3 h. Drug release was then obtained by NIR irradiation. Right: TEM (up) and SEM (bottom) images of the developed MNPs. Reproduced with permission from Ref. [201]. (**B**) SEM images of iron oxide mesoporous microparticles (left) and cells incubated with particles (right). Microparticles (indicated by red arrows) associated with breast cancer MCF7 cells, both as attached to the periphery, and internalized inside. Adapted and Reproduced with permission from Ref. [202] (**C**) Drug release from magnetized scaffold in response to macroscopic structural deformation. (**D**) A cylinder of a macroporous ferrogel reduced its height ∼70% when subjected to a vertical magnetic-field gradient of ∼38 A/m^2^, and SEM images of a freeze-dried sample in the undeformed and deformed states. (Scale bar: 500 μm.) Adapted and Reproduced with permission from Ref. [203] (**E**) SEM images of a magnetized scaffold made of a piezoelectric polymer, poly(vinylidene fluoride) (PVDF), and magnetostrictive particles of CoFe_2_O_4_. Preosteoblast cells were cultured for four days on scaffolds with different pore sizes. When magnetically stimulated, cells grew around the pores, probably guided by the magnetomechanical and magnetoelectrical stimuli. Adapted and Reproduced with permission from Ref. [240].

MCs at the nano- and microscale are useful for drug delivery, as conceived as injectable formulations and cell-interactive materials. For instance, drug-loaded MNPs can be systemically administered via intravenous injection and then magnetically guided to a target site through locally applied magnetic fields. Once at the desired site, the drug liberation can be triggered by different mechanisms. Shen et al. used NIR irradiation to release the antitumor drug DOX and the calcium ion channel blocker verapamil from injectable MNPs. The nanosystems were systemically administered and then targeted to the tumor by exposing them for a few hours to a magnetic field (about 2000 Oe) laid outside the skin in close proximity to the tumor (Figure 6A) [201]. Larger MCs realized at the microscale are also useful in drug release application, and their advantageous potential relies on two aspects. First, magnetic microsystems retain higher magnetization than MNPs, resulting in more controllable during remote navigation. The responsiveness to stationary magnetic fields of the magnetic microsystems is in general expected to be large enough to counteract the hemodynamic forces during circulatory distribution. Second, microsystems support higher payloads of bioagents and can optimally interact with cell membranes, adhering and adapting to the surface morphology of cells (Figure 6B) [202]. 

In addition to injectable formulation, the MCs can be used in targeted delivery as embedded in matrices that act as static platforms to control drug release or accumulation [203]. The MCs are typically used to enrich the matrices that serve as scaffolds for tissue repair [132]. The resulting nanocomposites can be magnetized under the application of magnetic fields and have increasingly been applied to tissue regeneration, especially for hard tissue repair [2,204]. This interest is justified by multiple reasons. In general, enriching the scaffolds with nanoparticulate material positively affects cell adhesion, viability, and proliferation [205]. This aspect is often reported for IONPs but not unique to them, as many other kinds of nanomaterials can provide topographical cues that modulate cell behavior [206,207,208,209]. However, using MNPs makes it possible to have remote control over cellular processes [131]. The dispersed MNPs entrapped in the scaffold matrix can be stimulated by a static or alternated external magnetic field, and the effects that such stimulation has on the cells can be different [131,138,210,211]. Magnetic cell stimulation is typically mediated by the action of mechanical forces that apply to the cell membrane and activate specific molecular signaling pathways in a process known as mechano-transduction [131,212]. For remote cell mechanostimulation through externally applied magnetic fields and magnetic nano-transducers, magnetic fields of moderate intensities (ranging from 1mT to 1T) are sufficient to trigger specific cell responses, which can be used to guide the process of tissue repair [131]. Common magnetic materials for tissue regeneration include: nanoparticles, polymers, bioceramic materials, and alloys [213,214]. Magnetic actuation of the scaffolds can improve their performance in tissue repair, impacting positively on new tissue formation. This especially applies to bone tissue engineering [213], in which novel methods combining scaffolds and stem cells with MNPs and magnetic fields have shown the ability to enhance cell osteogenic differentiation, as well as bone regeneration and angiogenesis by 2–3 folds over the controls [213,215,216]. In fact, magnetic actuation activates essential signaling pathways in osteogenesis, such as MAPK, integrin, BMP, and NF-κB. Augmenting bone repair and regeneration efficacy, magnetic actuation holds clinical potential for dental, craniofacial, and orthopedic treatments.

Although the magnetic scaffolds are proven to trigger osteogenic differentiation and therefore have been mostly involved in bone tissue engineering, they have also shown utility for the repair of other tissue types [137,217,218,219,220,221], and in relation to specific uses other than the direct modulation of cellular processes (e.g., bioimaging, cell patterning, robotics, drug delivery) [215,222]. In particular, magnetic actuation of scaffolds can serve to localize and control drug delivery [223]. Porous and fibrous scaffolds have been widely used to deliver drugs, proteins, and living cells for both tissue regeneration and cancer therapies [224,225,226,227,228]. However, in conventional scaffolds the release of bioagents is typically governed by the intrinsic chemical and structural properties of the matrix, that determine the kinetics of crucial processes, such as the diffusion of dispersed molecules, the scaffold material degradation, and the cell migration. Conventional scaffolds, therefore, do not permit dynamic external regulation of the topical therapy [224]. As a consequence, different routes to release biofactors on-demand from implantable scaffolds have been investigated [223,229]. In this context, magnetically driven approaches are emerging due to the favorable properties of magnetism, such as the remote controllability of materials, the high tissue penetration depth of magnetic fields, and the versatility of magnetic actuation mechanisms [213,216,230]. As an example, scaffolds can be enriched with magnetic material and loaded with biofactors. Then, externally applied magnetic fields can modulate the release of the biofactors via different mechanisms, which depend on the method for magnetic stimulation, the properties of the scaffold matrix, and the strategy of drug incorporation. If MNPs are stably conjugated to the scaffold matrix, they can induce physical deformations of the structure, so that the included substances are extruded. By subjecting MNPs to AMF, it is possible to locally increase the temperature, thus altering the structure of the surrounding matrix and the drug release kinetics. In such systems, bioactive molecules can be directly loaded into the scaffolds and conjugated to the polymeric structure. Alternatively, drugs can be loaded onto the MNPs which are then incorporated into the scaffold. When a magnetic field is applied, the MNPs vibrate detaching the coupled drugs and accelerating their release. 

Magnetic nanocomposites (especially in the form of hydrogels) are typically investigated as controllable drug delivery systems, although only a minority of them have been proposed for tissue regeneration applications. Magnetized scaffolds for tissue repair are typically loaded with factors that affect the three most crucial processes in implantology, namely tissue regeneration, graft vascularization, and fusion with the host tissue [224,231]. Growth factors are often applied to support these processes, as they play a pivotal role in directing regenerative pathways for many cell populations [223]. Magnetic nanocomposite scaffolds can be loaded with the growth factors that are necessary to stimulate cells over time, leading to complete biological and histomorphological tissue maturation. One of the first studies in this area was reported in 2011. Luo et al. loaded plasmid genes of the Vascular Endothelial Growth Factor (VEGF) onto chitosan/gelatin microspheres that were enriched with superparamagnetic magnetite crystals [206,208,224,232]. The magnetic microspheres were then incorporated into a scaffold for bone tissue repair. Oscillating and static magnetic fields induced local micro-movements of the microspheres, resulting in the release of the plasmids and transfection of the surrounding cells. The group demonstrated improved angiogenesis and osteogenesis within the scaffold, as well as enhanced scaffold vascularization and large bone defect repair. Magnetized scaffolds have been also proposed for wound dressing. In a recent study, itaconic acid was copolymerized onto starch and alginic acid in the presence of graphene sheets and magnetite nanoparticles [233]. The resulting magnetic composite hydrogel was loaded with guaifenesin to enhance wound healing. Guaifenesin could be released in a controlled manner by modulating the applied magnetic fields. 

Exposing MNPs entrapped into the scaffolds’ matrix to AMF causes the temperature to locally increase. The resulting thermal variations can affect the release kinetics of drugs loaded onto the scaffolds. For instance, when magnetite nanoparticles (NPs) were incorporated into PLGA scaffolds and then exposed to magnetic fields as per hyperthermia treatment protocol, heat was generated [234]. The heat varied proportionally to the concentration of MNPs and affected the release of minocycline, a drug for the treatment of glioblastoma. At temperatures close to the polymer’s glass transition temperature, the polymer’s chain becomes more mobile resulting in enhanced drug diffusion out of the scaffold. The same paradigm was adopted by Amini et al., who proposed magnetized nanofibers as a device to be implanted in the proximity of bone tumors for topic cancer therapy. In this system, magnetic bioactive glasses and the antitumor drug cisplatin were loaded onto chitosan-grafted PCL nanofibers [126]. These fibers were used to test the efficacy of the simultaneous application of chemotherapy and hyperthermia on MG-63 osteosarcoma cells. While no initial burst release of cisplatin occurred from the fibers, the release rate was accelerated under increased temperature (43 °C) as compared with the physiological condition. 

Controlling drug release from scaffolds is crucial for tissue regeneration. In fact, many tissue engineering approaches aim to recapitulate the sequences of events that drive tissue development in nature, and that are regulated by growth factors. For example, the sequence and timing of delivery of growth factors play a pivotal role in bone regeneration [235]. Although various biomaterials that deliver multiple factors have been presented, there still exists a strong demand for strategies to control the sequential drug delivery and replicate the key steps of tissue development to improve regenerative outcomes. To address this issue, Madani et al. presented a bone tissue regenerative biomaterial that could rapidly recruit stem cells and expose them to the Bone Morphogenetic Protein 2 (BMP-2, a potent inducer of osteogenic differentiation) with programmed timing [236]. BMP-2 can inhibit cell proliferation, resulting in a poor cell population of the scaffold. Therefore, BMP-2 has to be provided only when the scaffold has intensely been invaded by cells and a robust cell population is established. To delay the BMP-2 release after implantation, the group proposed a biphasic biomaterial that consisted of an outer porous gelatin compartment and an inner ferrogel compartment. The outer layer served to harbor stem cells and was therefore loaded with a stem cell recruitment factor, the stromal cell-derived factor 1-α, (SDF-1α). The core of the system contained the BMP-2. By applying external magnetic fields (0.56 T), the BMP-2 could be released from immediate to a delay of up to 11 days after implantation.

In 2011, Zhao et al. described a porous magnetic scaffold that could macroscopically deform under the application of an external magnetic field, and they proposed that the volume variation could serve to extrude different bioagents (Figure 6C,D), including mitoxantrone, plasmid DNA, and a chemokine from the scaffold [203]. Moreover, the scaffold could serve as a depot of various cells, whose release can be magnetically controlled. More recently, hollow fibers composed of alginate and enriched with IONPs were prepared by coaxial 3D printing [130]. The fibers also encapsulated drugs, proteins, or living cells in the core, in which a lower alginate concentration was used. When an external magnetic field was applied, the resulting scaffolds deformed, causing the various biofactors to be extruded. A formulation consisting of 10 wt.% of alginate and 13% of MNPs displayed repeated on-demand release capability in vitro and in vivo under intermittent magnetic stimulation. Cells loaded onto magnetized scaffolds can also be stimulated by the magnetoactive components of the substrate (Figure 6E) [237], and be then released in the proximity of the scaffold. This perspective holds promise for future drug-free strategies to control cell therapy in vivo. 

Even if intense efforts have been devoted to magnetic targeted drug delivery, fixing drugs after targeting is also extremely important in local therapies, such as tissue implantation. Research has therefore intensified on devices that guarantee the residence of delivered bioagents to the target site. A wearable drug fixation device was presented for cartilage repair via stem cell therapy [238]. An array of permanent magnets was mounted on a wearable band capable of wrapping the region of interest. The magnet configuration was determined through univariate search optimization and 3D simulation. Yielding a strong magnetic flux density (>40 mT) at the target site, the band could immobilize magnetic carriers at the cartilage defect sites in a rat model. In particular, the system’s retention action was tested on a stem cell spheroid made from cells labeled with MNPs, and a micro-scaffold composed of PLGA functionalized with MNPs via amino bond formation. The next research steps in magnetic therapy fixation are expected to generate devices matching the criteria for clinical translatability. 

Even in the absence of magnetic fields, doping the scaffolds with MNPs can alter their activity as a drug delivery system. For instance, the addition of MNPs reduced the degradation of silk fibroin scaffolds that have been proposed as multifunctional composite for tissue engineering [239]. In particular, the degradation rate of these sponges was strongly affected by the presence of the MNPs and correlated with the availability of active sites for proteases’ binding. First, hydrogen bonds formed between fibroin and MNPs, which strengthened the matrix structure. In addition, the complexation of iron atoms and tyrosines decreased the activity of hydrolases. These two processes synergized to delay the degradation of the system, in which cell viability was preserved.

In summary, magnetized scaffolds can be used not only to control the release of pharmaceuticals via different mechanisms (such as thermo-responsive disassociation and physical extrusion via structural deformation), but they can also serve to modulate the behavior of loaded cells, and also to affect the kinetics of material degradation, thus prolonging the permanence of bioagents in the desired site. 

## 4. Magnetic Guidance 

Magnetic materials and forces enable us to target different classes of therapeutic agents to the site of disease, feeding the interest in developing approaches that can overcome the inherent limitations in human use. One of the main challenges in the magnetic targeting of bioagents is that it limits targeting in deep tissue in human subjects, therefore making the translation into clinic arduous. Nevertheless, in recent years, novel approaches have emerged as a clinically viable solution for the site-specific delivery of small-molecule pharmaceuticals, biotherapeutics, and cells in patients. For example, the effects of deep-penetrating uniform magnetic fields can be exploited to accurately provide antirestenotic therapy to vascular targets and prevent injury-triggered re-obstruction of stented blood vessels [241]. The magnetization and guidance of cells can serve for different applications, including: magnetophoresis, cell-sorting, tissue engineering, and cell vectorization to target sites in vivo. Magnetic materials and forces can also control the motion of dynamic devices at the microscale, called micro-robots. In the following paragraphs, we will describe the use of magnetic forces to control the movement of cells and microrobots in different environments, highlighting their potential in biomedical applications.

### 4.1. Magnetic Guidance of Cells

#### 4.1.1. Magnetic Cell Manipulation 

Up to several million MNPs can concentrate within endosomes and lysosomes of labeled cells, rendering these cells responsive to inhomogeneous magnetic fields. MNPs can therefore mediate controlled and contactless movements of cells and subcellular structure. Cell magnetophoresis describes the motion of cells, which is caused by their loading with magnetic materials and their subsequent exposure to a nonuniform magnetic field created either by electromagnets or a permanent magnet [242]. In the last two decades, magnetophoresis of cells has been investigated for applications like cell separation, localized cell therapy, and tissue engineering. Within a unidirectional magnetic field gradient, the labeled cells experience a magnetic force, which can be defined as M(B) ΔB, where M(B) is the magnetic moment of the cell in the field B and ΔB is the magnetic field gradient. The magnetic moment of the cell can be approximated as the magnetic moment of one MNP multiplied by the number of MNPs per cell. The magnetic force that the cell experiences can drag it to a precise direction, but it is counteracted by several factors, like adhesivity of cells to substrates, the architecture of surroundings (e.g., porous or compact matrices), and viscosity of the medium. For cells suspended in liquids, the viscous force is defined as 6πηRV, where R is the cell radius, V is the cell velocity and η is the viscosity of the medium. Magnetophoresis of cells within more complex 3D environments (like tissues, scaffolds, or hydrogels) is also strongly affected by the physicochemical features of the embedding matrix. 

The magnetic fields generated by permanent magnets typically vary in the range of 10–50 T/m over a distance of approximately 1 cm. Labeled cells with an average iron load of 5 to 15 pg/cell therefore experience magnetic forces that range from 1 pN to a few nN. It is possible to deduce the iron load through magnetophoresis by determining the cell radius and velocity from video-microscopy. To label the cells with MCs in a quantifiable and reproducible way, the MNP formulation must be stable in the conditions selected for cell incubation. Importantly, magnetophoresis can detect potential nanoparticle aggregation and non-specific particle attachment to the cell membrane. If the cells are covered with particle aggregates, the outcome of the magnetophoretic assay will not lead to calculating the correct value of the intracellular iron load, as the obtained value will be higher due to extracellular aggregate pods. Magnetophoresis will instead reflect the cell velocity that is due to both internalized and membrane-attached nanoparticles. Therefore, as an iron dosage method, single-cell magnetophoresis allows one to identify potential artifacts that alter the quantification of iron, which is internalized by the cells [243]. Moreover, magnetophoretic cell sorting can serve multiple purposes, such as: (i) obtaining a fraction of labeled cells with a precise and homogenous iron load; (ii) separating magnetic cells from complex mixtures; or (iii) sorting them according to their endocytosis ability.

In 2009, Wilhelm et al. demonstrated the ability to control cell migration with external magnetic forces [244]. In this study, the magnetic field created by a thin magnetic tip, with forces as low as 30 pN, was sufficient to control the movements of aggregated and magnetized cells from the early developmental stage of *Dictyostelium discoideum*. The effects of magnetotaxis on cells in the presence of chemical gradients were studied in a competitive assay, where cAMP was slowly injected through a micropipette. Cells initially aggregated around the pipette, but when the injection was terminated, the cells were found to move towards the magnetic tip forming streams that were observable under the microscope. 

**Figure 7:**
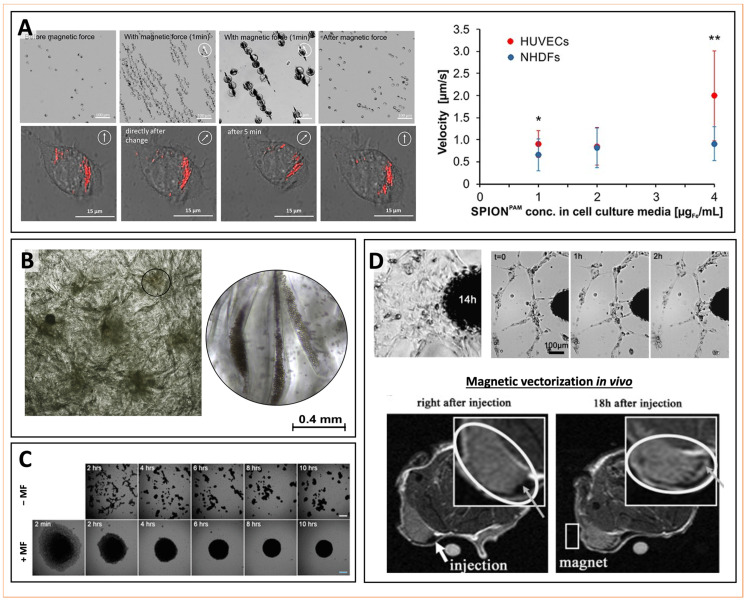
**Magnetic guidance of cells**. (**A**) Magnetic guidance for the formation of aligned cell clusters that are largely reversible upon removal of the magnet system. Magnetically induced velocities of SPION-labeled fibroblasts, endothelial cells, and cell clusters, guided on a square path by changing the direction of the quadrupole. Statistical significances between SPION-loaded HUVEC and NHDF cells are indicated with * and **. The respective confidential intervals are *p* ≤ 0.05 and *p* ≤ 0.0005, respectively, and were calculated via Student’s *t*-test. Confocal laser microscopy of macrophages labeled with fluorescent SPIONs guiding the intracellular organization of SPION-loaded endosomes upon varying magnet orientations. Adapted and Reproduced with permission from Ref. [245]. (**B**) Photograph of a scaffold seeded with magnetic Mesenchymal Stem Cells (MSCs), under the influence of the magnetic fields created by the tips. Magnetic fields induced precise cell patterning creating hexagonal multicellular assemblies within the scaffold. Adapted and Reproduced with permission from Ref. [251]. (**C**) Formation of pre-adipocyte cell spheroids in the presence (+MF) and absence (-MF) of an applied magnetic field over time (scale bars: 300 μm). Adapted and Reproduced with permission from Ref. [108]. (**D**) Top: endothelial cells magnetized with MNPs form dense tissue around the magnetic tip 14 h after seeding onto Matrigel, and ex novo formation of tubular structures connecting to the magnetic tip in 2 h. Bottom: MRI of in vivo guidance of magnetized endothelial cells injected into Matrigel, implanted subcutaneously in mice. After 18 h of application of gradient magnetic field, negative enhancement is diluted on Matrigel submitted to the magnet, whereas controls still show a localized area of hypointense signal (not shown). Adapted and reproduced with permission from Ref. [259].

Blümler et al. presented a system of permanent magnets in a cylindrical arrangement, which allows not only for steering the SPIONs on arbitrary trajectories but also for dragging labeled cells in suspensions along with predetermined directions (Figure 7A) [245]. Control over the movements of intracellular structures within individual cells was also demonstrated. In this work, the researchers combined a strong, homogeneous dipolar magnetic field with a constantly graded quadrupolar field, which was superimposed on the first. Whereas the homogeneous field aligned the nanoparticles’ magnetic moments, the quadrupolar gradient field exerted a force on the particles, predominantly guiding them with constant force and in a single direction. In a setup with a coaxial arrangement of two Halbach cylinders, the force direction could be simply adjusted by varying the angle between quadrupole and dipole. Furthermore, the force strength can be additionally scaled by adding another quadrupole, even doubling that of a single quadrupole upon mutual field rotation. The authors showed that magnetized macrophages could form needle-shaped aggregates of cells containing intra- and extracellular SPIONs, and that this aggregation tendency increased while increasing SPION-loading per cell, and can be reversed by removing the magnetic fields. Long pole-ladder-like structures might be useful to model or engineering structures with unidirectional cellular growth, having potential for research in cellular barriers, capillaries, and neural communication pathways [246]. Cells labeled with SPIONs were placed in the magnetic arena and then guided on a square path by changing the direction of the quadrupole by 45° every minute. This caused the cells to move at twice that angle. The direction and velocity of cells were analyzed based on the recorded videos, proving that labeled cells have increased magnetically induced average velocity that depends not only on the SPION concentration but also on the cell type. Moreover, SPIONs inside the cells strictly followed the dipole’s rotation, and the stirring effect could move the SPIONs-loaded endosomes inside the cells. Guryanov et al. used polyelectrolyte-stabilized IONPs to label neuronal progenitor cells and neurons differentiated from fibroblast-derived induced pluripotent stem cells. Then, they spatially guided these cells by using a permanent magnet, opening a new perspective on the future cell therapy for the treatment of neurodegenerative diseases [247]. In this regard, Marcus et al. published a thorough study on the optimal type and concentration of particles, as well as optimized incubation time, for magnetic manipulation of neuronal cells. Uncoated maghemite IONPs were efficiently internalized in neurons, without reported cytotoxicity. Furthermore, the MNP amount, which increased with the incubation time, reached a plateau after 24 h of exposure [248]. 

Magnetophoresis of cells can also be used to characterize and separate cells for biochemical analysis. For instance, this technique enabled the differential migration of red blood cells exposed to a high magnetic field, based on the content of deoxy and methemoglobins, which have paramagnetic properties as contrasted to the diamagnetic character of oxyhemoglobin [249]. Having a high hemoglobin concentration, human erythrocytes were ideal candidates to measure their migration velocity in cell tracking velocimetry. Under a magnetic field of 1.40 T and a gradient of 0.131 T/mm, erythrocytes with 100% deoxygenated hemoglobin or methemoglobins displayed similar magnetophoretic mobility of 3.86 and 3.66 × 10^−6^ mm^3^ s/kg. In contrast, the magnetophoretic mobility of oxygenated erythrocytes (from −0.2 to 0.3 × 10^−6^ mm^3^ s/kg) revealed a significant diamagnetic component relative to the suspension medium. 

In 2009, mesenchymal stromal cells from human bone marrow were magnetically targeted to fragments of degenerated human cartilage in vitro. Briefly, cells were injected into tissue culture flasks, in which osteochondral fragments were attached to the sidewall, and a magnetic force was applied for 6 h to the direction of the cartilage, resulting in the formation of a cell layer inclusive of extracellular matrix on the degenerated cartilage, suggesting that the method has applicative potential in tissue regeneration [250]. 

Magnetic cell manipulation has also several other applications in tissue engineering. Engineered tissue grafts are typically generated from biocompatible porous or fibrous scaffolds that are seeded with cells. Local seeding of cells in 3D scaffolds can result in inhomogeneous cell dispersion over the construct volume, but magnetic field gradients can drag magnetized cells through the constructs to distribute them more homogeneously. In addition, magnetic attraction can serve to induce, through magnetic tweezers, a defined 3-D cellular patterning. Damien et al. labeled mesenchymal stem cells with anionic MNPs and applied magnetic micro-manipulation not only to enhance the cell seeding process into pullulan/dextran scaffolds, but also to confine the cells into specific positions to achieve a well-defined spatial cellular organization (Figure 7B) [251].

Alternatively, magnetized cells can be manipulated for scaffold-free 3D cell culture systems [252]. In this case, the process of cellular assembly in multicellular aggregates occurs via magnetic levitation of labeled cells within the culture environment. This biofabrication technique is based on the ability of the magnetic force acting on the cells to be equilibrated with the gravitational force. Optimal levitation of cells leads to the aggregation and condensation of cells, which then form complex 3D cellular structures (Figure 7C) [108]. Magnetic levitation enables the production of spheroids with higher densities and more symmetrical structures than those obtained from traditional techniques (like the centrifugation method) [108]. Moreover, by differently patterning the magnetic fields, it is possible to modulate the morphology of cell constructs, and also to improve the fusion among spheroids, opening perspectives in creating larger and more complex structures in tissue substitutes [253]. Magnetic cell levitation is typically performed by labeling the cells with MNPs, and scaffold-free tissue constructs with different morphologies have been generated in this way from endothelial cells [254], fibroblasts, preadipocytes [108], stem cells, muscle cells [255], epithelial cells [256], tumors [257], and others. 

Contactless magnetic assembly of cell clusters has been primarily proposed to bypass the need for artificial scaffolds. Nevertheless, magnetic levitation can also apply to magnetized matrices. For example, Souza et al. levitated human glioblastoma cells within magnetized phage-based hydrogels consisting of gold nanoparticles, MNPs, and filamentous bacteriophage. By spatially controlling the magnetic field, the authors modulated the geometry of the mass of seeded cells and also obtained multicellular clustering of different cell types [258]. 

When submitted to remote magnetic forces, the cells suspended in liquids can move more freely than those that are adherent to substrates or those embedded in viscous or more solid matrices. Adherent and embedded cells cannot move if the magnetic forces do not overcome the adhesion constraints or if physical entities (like solid structures of the matrix) obstruct the movement. In such cases, the magnetic force acts on the organelles (i.e., endo-lysosomes) that have internalized the MCs. Wilhelm et al. demonstrated that magneto-manipulation of physically constrained cells allowed for the formation of vascular networks within an ECM-like matrix (Matrigel) [259]. Briefly, endothelial progenitor cells were labeled with uncoated anionic iron oxide nanoparticles, seeded on Matrigel, and magnetically manipulated at a distance by applying a magnetic field gradient that served to pilot different phases of tubulogenesis. In the first 14 h after seeding, cells migrated towards the magnetic tip to form dense tissue just around and below the tip (Figure 7D, top left). After 18 h, the magnet tip was applied, and local connections of vascular tubes started to form towards it. Lines of cells perpendicular to the direction of the magnetic tip were deformed due to the attraction force to the tip (Figure 7D, top right). In addition, the magnetic guidance of cell homing was demonstrated in vivo. Labeled cells were injected into matrigels implanted in subcutaneous pockets on the flanks of mice. A permanent magnet was placed on the opposite side of the injection site, resulting in a magnetic field gradient that guided the cells towards the magnetic pole, as shown by the elongated hypointense signal detected by MRI (Figure 7D, bottom). 

This experiment was an example of vectorizing magnetic cells in vivo following their topical injection within tissue or tissue substitutes. Nevertheless, in vivo cell targeting experiments are typically performed by releasing the magnetized cells into the systemic circulation, before applying a magnetic gradient that guides or retains them to the desired locations. In the following section, we will survey the use of MCs to direct cells to a specific target after their injection into the circulatory system. 

#### 4.1.2. Magnetic Targeting of Cells In Vivo

The successful outcome of cell-based therapies primarily depends on the effective cell delivery to the target site. As certain lesions are located in sites that are difficult to reach (such as the spinal cord and the joints), novel strategies to localize and maintain the position of curative cells are demanded. Magnetic vectorization of cells in vivo is an emergent approach that holds promise for cell therapy, regenerative medicine, and tumor treatment [260]. Briefly, cells with a therapeutic function are labeled with magnetic materials (like the MNPs) and injected into the circulation of living beings. Then, a static magnetic field is used to retain the cells within specific locations in the body. Magnetic targeting can promote more effective or focused cell delivery to areas that cells usually do not reach after systemic administration. Being compatible with use in biological systems and noninvasive control of cells in vivo, magnetic cell targeting is an attractive solution to improve the localization and the beneficial effects of stem cells used for tissue regeneration, as well as immune cells exploited for tumor treatment [261]. 

For magnetic cell targeting in vivo, cells must be loaded with MNPs following some general observations. The labeling procedure must ensure that cell viability and functions are preserved, but also that a sufficient amount of particles are internalized. Whereas MNPs with large size (>200 nm in diameter) and a neutral charge are readily internalized by macrophages, the particles with small size (<200 nm in diameter) and positive or negative charges can be efficiently taken up by nonphagocytic cells, like the stem cells [262]. Several reports of stem cell magnetic targeting have used MNPs with sizes ranging between 20 and 100 nm, which is considered as an optimal size range for uptake by stem cells. Nevertheless, it was also demonstrated that larger MNPs (with a diameter above 0.9 μm), if positively charged, can also be well incorporated by both cardiac-derived stem cells and endothelial cells, and then used for magnetic targeting in vivo [263,264,265,266]. 

Another fundamental requirement in magnetic cell targeting is that the MNPs are noncytotoxic at the needed concentrations. The toxicity of MNPs strongly depends on the physicochemical profile of the particles, which accounts for the type of coating, the tendency to aggregation, and the overall stability. Moreover, other factors that define the interaction of the MNPs with the biological systems contribute to the toxicity of the particle formulation (e.g., pathways of internalization and stability within the cells, as well as their interaction with membranes and cytoskeleton). It has to be noted that the suitability of MNPs for magnetic targeting is not directly related to their performance as imaging agents, namely as contrast agents for MRI or other imaging technologies. As an example, the commercial formulation “FluidMAG” performs better than Ferumoxides (Feridex in the USA, Endorem in Europe) as a cell-targeting vector, since FluidMAG is better taken up by cells and displays improved magnetic properties helping the spatial guidance [267,268].

Once the cells are labeled and injected into the living system, their localization to the desired region occurs in response to the interaction between their magnetic cargo, and the magnetic field which is applied. Most studies have used externally placed magnets, including permanent magnets [259,264,265,269,270,271,272], electromagnets [250,273], and even MRI systems [267]. Some in vivo studies of magnetic cell targeting are listed in Table 1 and categorized in relation to the target organ.

One typical application is to guide transplanted stem or progenitor cells to the injured tissues, where they are held by the magnetic fields [260]. Once in the lesion site, stem cells can restore the damaged cell population by differentiating into specific cell types or by acting as localized immune modulators [299,300]. In particular, stem cells can secrete vesicles and soluble factors with immunoregulatory or reparative effects. Kobayashi et al. investigated the opportunity to accumulate Mesenchymal Stem Cells (MSCs) in osteochondral defects of the patellae in rabbits and pigs after intra-articular injection [286]. In the rabbits, the magnetized cells localized to the osteochondral defect when exposed to an external magnetic field, as shown by macroscopic and histological observation. In the pigs, cells attached to the chondral defect were observed arthroscopically. By proving that labeled MSCs can be magnetically guided from a local injection site to the desired place in the knee joint, the authors proposed that the magnetic targeting of locally injected therapies is applicable to repair the human cartilage defects caused by osteoarthritis or trauma by means of a scarcely invasive technique [219,286,301].

Magnetic cell targeting has widely been investigated for applications within the cardiocirculatory system. For example, it was used for intracoronary delivery of cardiosphere-derived cells or endothelial progenitor cells in a rat model of acute ischemia/reperfusion [265,272]. In these studies, magnetized cells were guided through permanent magnets to the heart tissue. The magnetic setup used by Cheng et al. relied on an actual flux intensity of 0.3 Tesla that was generated by a 1.3 T magnet placed ~1 cm above the heart [264,265]. A four-times greater cell retention was observed in the hearts of animals receiving the magnetically targeted cell therapy. Chaudeurge et al. implanted a 0.1 T magnet into the subcutaneous layer, producing an actual working intensity that was far less than 0.1 T. Magnetic targeting at these conditions failed to augment the cell retention as quantified by Reverse Transcriptase-Polymerase Chain Reaction (RT-PCR) [274]. Another group showed that cell capture efficiency positively related to the magnetic flux density between 10 and 640 mT, reaching around 90% of cell retention with a magnetic flux density of 640 mT, a magnetic intensity gradient of 38.4 T/m, and a flow velocity of 0.8 mm/s [278].

Riegler et al. targeted magnetized MSCs in a rabbit model of vascular injury treated with interventional balloon angioplasty [268]. By using a clinically applicable permanent magnet, it was possible to increase cell localization up to six folds as compared to the controls. Augmented cell retention led to a reduction in restenosis three weeks after cell delivery [268]. Magnetic targeting of mesenchymal stromal cells (MSCs) in the lungs was recently investigated [302]. In this study, MSCs were made magnetically responsive by incubation with citrate functionalized magnetic nanoparticles. After being intravenously injected into murine models of experimental silicosis, the magnetized cells were retained in the lungs with the aid of a pair of circular magnets attached to the chest of each mouse through a surgical tape jacket. The higher fraction of retained cells was associated with enhanced beneficial effects for the treatment of silicosis in mice, holding promise for the cure of chronic lung disease. Magnetic cell targeting in the respiratory system has not been well explored, as most cells that are administered systemically might remain trapped in the narrow capillaries of the lungs. In this study, the technique was investigated in the perspective to rather prolong than improve the MSC retention on site. Nevertheless, the investigations were inconclusive in explaining how the cells are cleared from the capillaries, a process that remains incompletely understood.

**Figure 8:**
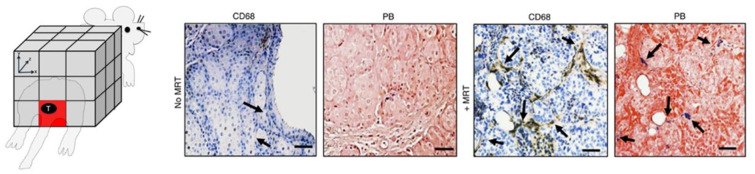
**Magnetic Resonance Targeting technique.** Magnetic resonance targeting (MRT) to vectorize circulating macrophages to prostate tumors and lung metastasis. Schematic of targeted regions using imaging gradients for MRT, where a–*y* gradient was equally applied across the animal to target the location of the prostate as depicted (red box). Histological staining of paraffin wax-embedded tumor sections with an anti-human CD68 antibody and Prussian blue (CD68-positive macrophages are brown and SPIO-positive macrophages are blue: see arrows). Adapted and Reproduced with permission from Ref. [295].

Magnetic targeting can be followed by the imaging technologies that are typically used for the visualization of MCs in vivo, such as MRI. Intriguingly, clinical MRI scanners are able not only to track the location of magnetized cells but also to act on the process of the targeted vectorization. This integration between imaging and guidance is referred to as *magnetic resonance targeting.* In 2010, Riegler et al. applied magnetic field gradients inherent to the MRI system, to steer ferromagnetic particles to their target region [267]. This work proved the feasibility of imaging-integrated magnetic cell targeting in a vascular bifurcation phantom while varying the flow rates and testing different types of particles. In conclusion, 75% of labeled cells could be guided within the vascular bifurcation. Muthana et al. labeled macrophages with SPIONs and then apply pulsed magnetic field gradients to guide the magnetized cells to tumors and metastatic sites in vivo [295]. This way, the authors steered the circulating macrophages from the bloodstream to the tumor site and the lungs in orthotopic prostate xenograft murine models. The enhanced macrophage tumor infiltration was accompanied by a reduction in the tumor burden and metastasis to the lungs (Figure 8), being promising for future theranostic development of magnetic cell guidance. 

Other examples of immune cells that were magnetically targeted against the tumors include cytotoxic lymphocytes and natural killer cells (Table 1). These reports demonstrated that systemically administered immune cells can be retained at the tumor and metastasis sites upon magnetic attraction. 

Although magnetic cell retention is very promising for cell therapy, to date it is still uncertain if magnetic forces can efficiently allow the cells to reach the target site in many applications. In fact, for successful delivery, magnetic forces then need to overcome the force that opposes the movement of cells under guidance. When cells are systemically injected, the magnetic drag has to overcome the force of the bloodstream flow. When cells are locally transplanted into the tissue, the magnetic drag must exceed the physical hindrance of the materials that build up the matrix surrounding the cells. Moreover, after engrafting to the target site, the cells might not persist in that location after removing the magnetic forces. Therefore, magnetically guided systems endowed with intrinsic mobility are promising to develop effective magnetic guidance of cell therapy, as well as drug release. The next section describes dynamic devices under magnetic guidance that have the potential to improve the efficacy of magnetically targeted therapy.

### 4.2. Magnetic Guidance of Microrobots 

The MCs can be used to confer controllability to microscaled materials that are endowed with intrinsic motion abilities or designs that improve motion dynamics. Such devices referred to as microrobots, are typically involved in biomedical applications, due to their size, which renders them effective to interact with cells and tissues, and move through anatomical structures. In microrobotics, magnetic actuation provides the intrinsic advantage of using magnetic fields, which are safe for biological tissues and can penetrate them. Moreover, as microsystems have limited space for onboard energy storage, magnetic fields are also advantageous as they wirelessly transfer actuation power to the microrobots.

Microrobots based on MCs can be classified into two major classes based on their composition: purely synthetic microrobots and bio-hybrid robots. Purely synthetic microrobots are made of synthetic materials only, and their actuation results from a combination of applied magnetic forces and propellant designs. Bio-hybrid robots are robots that contain a biological living component [303,304]. In bio-hybrid robots, this component is represented by cells endowed with self-propulsion ability. In the next paragraphs, we will describe these two main categories of magnetic microrobots.

#### 4.2.1. Synthetic Microrobots

Synthetic microrobots use the MCs as a platform to release a therapeutic cargo to a specific destination. Magnetic microrobots can be guided to the target sites through contactless magnetic control, and deliver bioagents like cells or drugs. The microrobot body can be made of hard or soft magnetic materials [305]. Microparticles made of NdFeB, SPIONs, CrO_2_ powder, and FePt nanoparticles are the most typically used materials, but also other magnetic micro/nanoscale entities (such as molecules, discs, or wires) can be embedded inside the robot, absorbed, or sputtered onto the robot to form a nanofilm coating [305].

**Figure 9:**
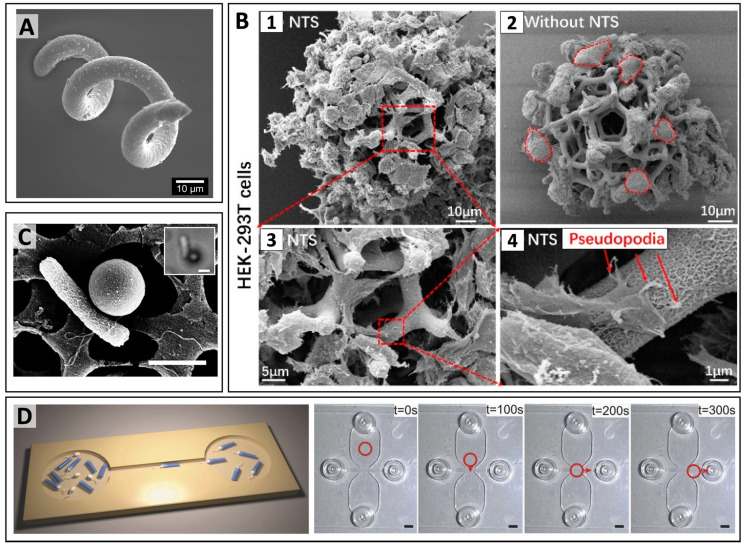
**Magnetic microrobots.** (**A**) SEM image of Spirulina-templated magnetic helical microswimmers. Adapted and Reproduced with permission from Ref. [308]; (**B**) Cell-carrying magnetic microrobots with bioactive nanostructured titanate surface for enhanced cell adhesion. SEM image of HEK-293T cells on microrobot after 1 day of incubation with (B1) and without (B2) nanostructured titanate surface; (B3) high-magnification SEM image of HEK-293T cells on microrobot with NTS; (B4) High-magnification SEM image of single HEK-293T cell with protrusive pseudopodia on microrobot with NTS. Adapted and Reproduced with permission from Ref. [325]; (**C**) Bacteria-driven microswimmers for targeted active drug delivery. Adapted and Reproduced with permission from Ref. [330]; (**D**) Magnetic guidance of microrobots based on sperm cells from a chamber containing a mixture of microtubes and sperm cells (left chamber) into a clean chamber (right) of an ibidi-chip. Scale bar: 3 mm. Adapted and reproduced with permission from Ref. [332].

Using microrobots for biomedical applications requires locomotion in fluids. Microrobots that can controllably move in liquid environments have been designed and developed by mimicking the natural locomotion of the swimming microorganisms [306]. These propulsion strategies have been of great inspiration to realize specific types of locomotion under magnetic control [306]. As an example, micropropellers with a helical design are inspired by bacterial flagella (Figure 9A) [307,308]. To drive helical microrobots, external rotating magnetic fields are typically used in order to avoid the need for an on–board rotary micromotor. In addition, the steering of these robots is achieved by varying the axis of rotation of the magnetic field, whereas the overall speed mostly depends on their helical shape [309]. Another bioinspired design for magnetic microrobots resembles the anatomy of sperm cells and other unicellular eukaryotes swimming through flexible flagella that are at least ten times larger and longer than bacterial flagella [310]. Such flexible filaments can propagate bending waves that provide propulsion to the whole cell body. Magnetic microrobots with flexible tails have been proposed, which move similarly to eukaryotic flagellated propellers [311,312,313,314]. For example, Pak et al. demonstrated a microrobot with a magnetic head and a flexible nanowire tail that can move in liquids as actuated by a magnetic rotating field [315]. Moreover, flexible structures with distributed magnetization can be used to achieve the propagation of bending waves leading to the undulation of artificial structures under magnetic actuation. Undulatory motion enabling locomotion in fluids can also be realized in the form of oscillating sheet-like structures [316,317]. One example is represented by milliscaled magnetoelastic robots [316,318]. By applying an external rotating magnetic field, a sheet with a periodic magnetization profile can be actuated in a way that propagating bending waves run along the axis of the magnetization gradient [319]. Magnetic flexible composites can be produced at the microscale as well if SPIONs are loaded onto the elastomeric structure [317]. 

MCs-based microrobots can be loaded with molecules, drugs, and cells. When reaching the target site, the delivery of the bioagents can occur via a passive or an active mechanism. In fact, magnetic microrobots can spontaneously release their cargo in response to a specific environmental condition. The most typical example is the pH variation, which is a hallmark of inflamed tissue or tumors. Chitosan hydrogels for instance have been proposed for conditional localized drug delivery upon acidic conditions, as they undergo physical deformations that can induce the release of loaded molecules [320]. Fusco et al. covered their magnetic microrobots with a chitosan gel layer and demonstrated the sustained and efficient release of a model drug [321,322]. The delivery of the payload from magnetic microrobots can also be controlled and triggered in response to an external trigger or an environmental cue [306]. For instance, thermoresponsive hydrogels containing MNPs can release molecules on demand from microrobots via heating by magnetic induction [323]. 

Magnetic microrobots can also be used to manipulate cells or carry them to a specific destination (Figure 9B) [324,325]. Breger et al. reported Poly(N-isopropylacrylamide) (PNIPAM)-based thermoresponsive microrobots with a gripper design [326]. They embedded iron oxide NPs into the porous hydrogel layer used to fabricate the microrobots, and showed that they could command them remotely through magnetic fields to actuate some conformational change. Through these movements, their microrobots could excise and capture cells from a live cell fibroblast clump. Microrobots are increasingly studied for their potential in the delivery of cells for targeted cell therapy. Li et al. proposed a magnetic microrobot for the delivery of stem cells [324]. They fabricated magnetic skeletons with burr-like porous spherical structures to improve the magnetic driving capability and maximize the cell-carrying capacity. Jeon et al. presented a helix-shaped microrobot where hippocampal neural stem cells could attach, proliferate, and differentiate into various mature neural phenotypes [327]. These microrobots were also loaded with MSCs derived from the human nose and then manipulated in vivo, inside the intraperitoneal cavity of a nude mouse. Go et al. used magnetic microrobots to control the localization and delivery of human adipose-derived MSCs for knee cartilage regeneration in a rabbit model [328]. The robot’s scaffold consisted of a spherical PLGA microstructure decorated with MNPs (Ferumoxytol), and the stem cells could grow over its surface. Following injection into the wounded-knee of the animal, the microrobots were actuated through an oscillating magnetic field. The robots accumulated in the target injured site within the knee, and then were retained in place through a permanent magnet. This way, the cells were facilitated in adhering to the surrounding tissue and proliferating there. Magnetically propelled spherical microrobots with burr-like designs have already been tested for transporting cells in different animal models [324]. For instance, MC3T3-E1 cells have been transported within the yolk of zebrafish embryos. Although the successful use of these cell-carrying machines in vivo was recently demonstrated [285,324], controlling them while they move noninvasively through the body to a target site is still an open challenge.

#### 4.2.2. Bio-Hybrid Microrobots

Robots with a biological living component that actively contributes to the movement of the whole assembly are referred to as bio-hybrid robots [304]. Bio-hybrid microrobots use natural biological actuation for propulsion. They integrate motile cells (such as bacteria, algae, sperm cells, or leukocytes), and are typically controlled via magnetic actuation [329]. Park et al. proved that multifunctional bacteria-driven MCs enable enhanced drug transfer as compared to passive microparticles (Figure 9C) [330]. The authors prepared stochastic microswimmers by loading *Escherichia coli* (*E. coli*) bacteria with polyelectrolyte-based DOX-loaded microparticles embedded with MNPs. The microrobots moved at average speeds of up to 22.5 μm/s, and their dynamic control was demonstrated by applying a chemoattractant gradient and a magnetic field to induce biased and directional motion, respectively. In another work, bio-hybrid microrobots targeting infectious biofilms were generated by combining the magnetotactic bacteria *Magnetosopirrillum gryphiswalense* with mesoporous silica microtubes loaded with antibiotics [331]. Through magnetic guidance, the particles were delivered to the matured E. coli biofilm, where they released the drug and caused the biofilm disruption. 

Microrobots based on sperm cells have been intensely investigated for their multifunctionality, actuation efficiency, and their controllability (Figure 9D) [311,312,332]. A magnetic bio-hybrid microrobot based on flagellate propulsion for drug delivery was developed by Xu et al. [333]. They created a sperm-hybrid micromotor consisting of a motile sperm cell that serves both as a propulsion source and a drug carrier. The synthetic component of the bio-hybrid was a 3D-printed magnetic tetrapod, namely a tubular microstructure made of a polymer and coated with 10 nm Fe and 2 nm Ti. The tetrapod could release the sperm cell in situ, as it consisted of four arms that could deform upon mechanical input. The arms could bend externally and open the tetrapod to liberate the sperm when the robot hit the tumor wall. This microrobotic platform was not only endowed with high drug loading capacity and self-propulsion, but it could also be magnetically guided to the target site and release its cargo upon mechanical trigger while exploiting the sperm penetration ability. 

The cellular component of the bio-hybrid microrobots contributes to their biodegradability and structural compliance, which helps for safe interaction with biological structures and circulation within living environments. Moreover, the small size and swimming abilities of the integrated microorganisms and motile cells turn helpful to efficiently navigate within biological fluids and/or through intricated anatomical structures (e.g., vessel networks), which are a prerogative for most biomedical applications, like targeted delivery and cell/tissue manipulation. These abilities combine with the capability of self-repair, flexibility, and even multiple degrees of freedom [334]. Thus, bio-hybrid robots at the microscale have a great potential for biological applications.

## 5. Imaging of Magnetic Micro/Nano-Systems 

**Figure 10:**
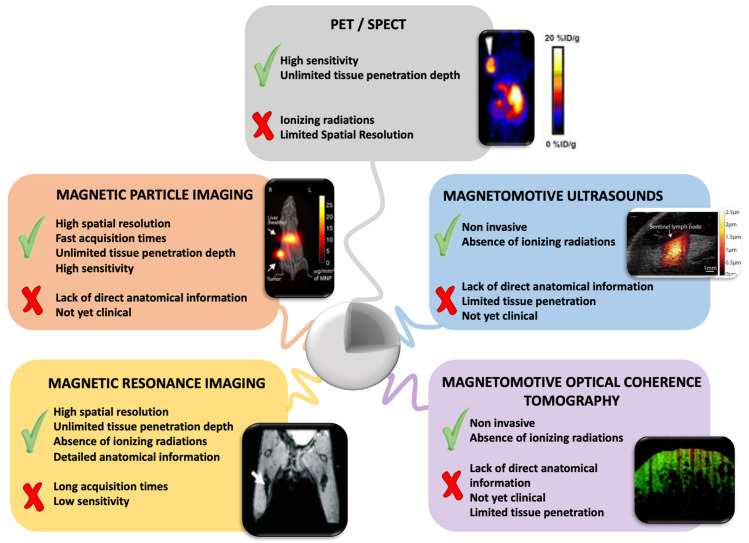
**Advantages and limitations of the imaging techniques for tracking of MCs.** MCs can be tracked by various imaging techniques (PET/SPECT; magnetic particle imaging, magnetomotive ultrasounds, magnetic resonance imaging, and magnetomotive optical coherence tomography). The selection of a precise technique is based on the assessment of the associated advantages and disadvantages. Adapted with permission from Refs. [364,365,366,367,368].

MCs can be visualized via various imaging techniques (Figure 10). This makes them extremely attractive as delivery systems, as they enable one to monitor the delivery of bioagents in real-time. In Section 5.1, the main imaging techniques allowing for the visualization of MCs will be presented, describing the basic principles and enabling technologies, as well as the properties that are needed in the MCs to be detected via a specific technique. In Section 5.2, we will discuss the opportunity to combine mechanisms of imaging and therapy within one MC and realize multifunctional platforms.

### 5.1. Imaging Techniques to Visualize MCs 

MRI is by far the most popular imaging modality to track magnetic systems. MRI uses powerful magnets and radiofrequency pulses to visualize soft tissues within the body. In principle, during an MR scan, a living subject is exposed to an intense magnetic field (1.5 or 3 T for clinical MRI; and 1, 3, 7, 9.4, or 11.7 T for preclinical MRI) that induces the alignment of magnetically active atomic nuclei in the matter [335,336]. In biological tissues, these nuclei are mostly protons (namely hydrogen nuclei) of fat and water molecules. Nuclei can be aligned parallelly or anti-parallelly to the magnetic field direction. The net difference in the number of nuclei aligning in the opposite directions is what generates the MR signal [337]. A pulsed radiofrequency can be then used to temporarily disturb the alignment distribution of the nuclei, which subsequently returns to the original state in a process known as relaxation. Relaxation occurs through two concomitant processes, termed longitudinal and transverse relaxation and characterized by specific kinetics. In these two processes, the time required to restore the equilibrium state is referred to as relaxation time, with T_1_ and T_2_ indicating the longitudinal and transverse relaxation times, respectively. The relaxation rates in the two processes depend on the physico-chemical environment surrounding the nuclei [338]. 

In MRI, the relaxation times are therefore assessed to gain information about the investigated matter. To increase the sensitivity of the technique, the relaxation processes can be altered by administering contrast agents, namely materials with the capability to vary the local magnetism experienced by the tissue nuclei. In particular, these compounds work by augmenting the longitudinal or transverse relaxation rates (R_1_ and R_2_) [339,340]. For instance, the MCs based on iron oxide predominately shorten the transverse relaxation time, therefore acting as T_2_ contrast agents [42,341]. Iron oxide-based MCs include: SPIOs (superparamagnetic iron oxide nanoparticles); USPIOs (ultrasmall superparamagnetic iron oxide nanoparticles); and MPIOs (microparticles of iron oxide). Although MCs are typically used to accelerate the transverse relaxation, some IONPs can also serve as T_1_ contrast agents, primarily reducing the longitudinal relaxation time [61,342,343,344]. The most commonly used MCs in the clinics are the USPIOs, which have typical diameters ranging from 20 to 50 nm and are characterized by a long circulation half-life. In contrast, as compared to USPIOs, MPIOs display a larger size and contrast-to-noise value per particle, and higher ligand valency, but also a shorter half-life (<5 min). As such, MPIOs are extremely suitable for cell labeling procedures (including in vivo labeling) but less performant in applications that require direct systemic administration of the particles [345]. In general, MCs for MRI are composed of a metallic core surrounded by a biocompatible coating, which varies in composition, total net charge, and surface functionalization. In micro/nanocomposites, the magnetic NPs can be loaded not only in the system’s core but also inside the shell. Detailed reviews about iron oxide-based MR contrast agents are available in the literature [346,347,348]. 

The main advantages of MRI are the absence of ionizing radiations, the high spatial resolution (preclinical: micrometers, clinical: ~1 mm), and unlimited tissue penetration [349,350]. The major limitation is a low sensitivity, which derives from the fact that the difference between the nuclei populations that, under the application of a magnetic field distribute in the two possible alignment states, is very small. The low sensitivity then makes it necessary to provide highly concentrated contrast agents (10^−3^ to 10^−5^ mol/L) [335,336]. In view of visualizing the bioagents delivery, an important consideration is that the MRI signal is not directly associated with the contrast agent itself, but it represents changes in environmental magnetic properties induced by the contrast agents. As such, this signal lacks information in terms of contrast agent localization at a high spatial resolution.

A more recent and likewise appealing technique is Magnetic Particle Imaging (MPI). MPI was firstly proposed in the early 2000s to track magnetic systems with an extremely high spatio-temporal resolution and without any background signal [351]. In MPI, a magnetic system (like a MC) is administered to a living subject exposed to a magnetic field similar to that used for MRI. However, in contrast to MRI, the magnetic systems themselves are detected, rather than the effects that they induce in their local environment [352]. Changing magnetic fields are applied to produce a single magnetic-field free region (the field-free point, FFP) in the imaged sample, and, if any magnetic material is located at the position of FFP, it will generate a signal. By steering this region across the sample, an image of the magnetic tracer is generated. As only information about the magnetic tracer is provided, anatomical images have to be co-acquired with a structural imaging technique (e.g., Computed Tomography (CT) or MRI) to precisely localize the MPI signal [353]. Nevertheless, MPI offers several advantages including high spatial resolution, fast acquisition times, unlimited tissue penetration depth, and high sensitivity [354]. As such, MPI is likely to become crucial for imaging MCs in the near future. MPI contrast agents are typically IONPs with size-dependent magnetic relaxation properties and designs that are tuned to maximize signal generation [355]. Available SPIOs are grossly inadequate for MPI and do not translate well in vivo. In fact, ideal MPI contrast agents should be extremely uniform in size (monodispersity of the core and hydrodynamic sizes of NPs), as for a given excitation field frequency there is a single particle diameter associated with a resonant, or perfectly in-phase, magnetization response for maximum signal intensity. For example, a diameter of ∼25 nm represents the optimum core diameter for a 25 kHz excitation frequency [356]. However, nanosystems were not the only systems to be developed for MPI. In fact, certain magnetic microcarriers have also found applications as MPI agents [357]. Zahn et al. designed magnetic microspheres with a size that could be tuned between 1 and 2 µm, which displayed MNPs onto the surface [358]. Co-loading a drug onto such microsystems opens perspectives in MPI-monitored targeted drug delivery.

Another emerging technology that uses MCs as contrast agents is magnetomotive ultrasounds (MMUs) [359]. MMUs use the vibration induced in the adjacent tissues by SPIONs, or other magnetic systems, subjected to an external magnetic field. MCs can be systemically administered to patients or living beings; then electromagnets, permanent magnets, or a combination of the two, are applied on the surface of the body. The MCs are displaced within the tissue through their immediate surrounding. When the magnetic field is removed, the MCs tend to return to their initial position [360]. This movement is then detected with ultrasound imaging by using a frequency- or time-domain analysis of echo data. MMUs can also be combined with photoacoustic (PA) imaging, a biomedical imaging technique that relies on the generation of sound waves as a result of light absorption [361]. These sound waves are then detected by ultrasound transducers and analyzed to produce images. PA and MMUs can be combined by merging IONPs with nanorods in larger MCs, with the advantage of PA background noise suppression [361].

Similar to MMUs, magnetomotive optical coherence tomography (MMOCT) exploits the movement of a magnetic contrast agent induced by an external magnetic field [362]. The resulting motion is then tracked by using low-coherence NIR light and interferometry. In comparison to MMUs, the penetration depth of MMOCT is limited [360]. 

Molecular imaging techniques using ionizing radiations, such as Positron Emission Tomography (PET) and Single-Photon Emission Computed Tomography (SPECT), can also be employed to track the delivery of MCs and the release of bioagents, but in such a case one needs to add a radioactive isotope to the MC [363]. The hybrid systems can be employed for multimodal imaging, with a wide range of applications.

### 5.2. Theranostic Applications of MCs

Since its first outbreak at the end of the 1990s, the word “theranostics” has become increasingly popular among scientists. This term derives from the combination of the words “therapy” and “diagnostics” [369,370], and refers to the use of systems that deliver an imaging and a therapeutic agent at the same time [371]. Theranostic systems can be exploited in a variety of fields [372], including: drug discovery [373]; real-time assessment of drug pharmacokinetics [374]; therapy screening/selection [375]; imaging drug delivery [376]; and imaging triggered drug release [377]. For these purposes, the contrast agents should be designed to combine capabilities of diagnosis and treatment in one platform. MCs are ideal candidates for such an application, as they can be used in MRI, MPI, magnetic targeting, hyperthermia, cell labeling, and controlled drug release. The following paragraphs briefly survey the use of MCs in different fields of theranostics.

#### 5.2.1. Magnetic Fluid Hyperthermia

Magnetic fluid hyperthermia (MFH) is a technique in which a biocompatible magnetic fluid suspension is locally or systemically administered and then heated up, above 37 °C. The temperature increase (to about 41–46 °C) causes cell apoptosis, and/or enhances the susceptibility of the target tissue to other therapies such as radiation and chemotherapy [378,379,380]. Heating can be induced through high intensity focused ultrasounds (HIFU), radiofrequency heating, microwave radiation, and laser photocoagulation [381], with high tissue penetration and specificity, and minimal heating of the surrounding tissues. The heating ability of the magnetic nanoparticles depends on their specific absorption rate (SAR), a property that is a function of particle size, shape, coating, surface charge, aggregation state, interaction with proteins corona, and even the dispersion medium [382]. The magnetic properties of the suspensions used for MFH allow for imaging during hyperthermia by both MRI and MPI. This enables the selective heating of pathological tissue, without affecting the organs where MCs tend to non-specifically accumulate (i.e., spleen and liver). In order to design the ideal nanoagent for simultaneous MRI and MFH, the T_1_ and T_2_ relaxivities and the SAR should be maximized [383]. For this purpose, iron/platinum (FePt) nanoparticles were combined with kaolinite and cetyltrimethylammonium bromide, and loaded with DOX, to serve as MFH agents. These nanoparticles presented a transversal relaxivity r_2_ of 29.32 mM^−1^ s^−1^ and a SAR of 36.91 W/g and were used as a multifunctional drug delivery system for MRI, magnetically guided targeting, and therapeutic treatment [384]. As compared to Fe_3_O_4_, metallic Fe, Au–Fe_3_O_4,_ and FeCo nanoparticles, the FePt nanoparticles for combined MRI and MFH, demonstrated higher chemical stability, and better performance as contrast agents for MRI [385]. Following stereotactical administration of IONPs in patients bearing recurrent glioblastomas, the particle distribution could be imaged by MRI and the heating boundaries could be estimated by applying a forward model [386]. The combined MFH and radiation therapy significantly increased the overall survival of patients [386]. 

Zahn et al., instead, prepared PLGA microspheres loaded with magnetic multicore NPs and the anticancer drug Camptothecin to investigate the hyperthermia-induced drug release [357]. SAR measurements showed a promising heating power of the microspheres (161 W/g) that led to the release of around 80% Camptothecin at 43 °C. Similar results were obtained by Zhang et al. who used nano-in-micro thermo-responsive microspheres loaded with Methotrexate or 5-Fluorouracil [387]. Stocke et al. developed inhalable magnetic nanocomposite microparticles for targeted pulmonary delivery via spray drying [388]. This magnetic nanocomposite displayed excellent thermal properties (SAR > 200 W/g), holding promise for innovative bronchial lung cancer treatment.

**Figure 11:**
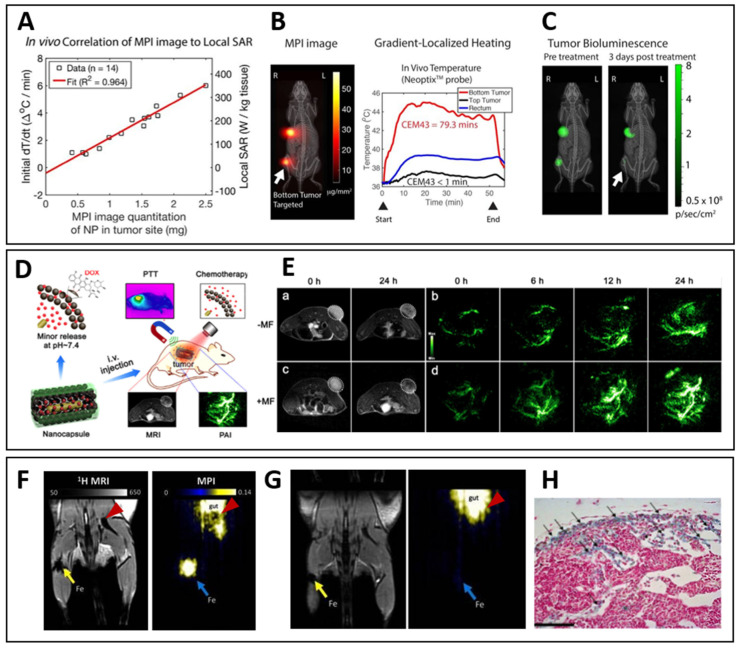
**Imaging MCs in theranostic applications.** (**A**–**C**) In vivo localized MPI guided MFH experiment. (**A**) MPI images provided quantitative information of SAR estimate at the tumor site, (**B**) MPI image of the dual tumor MDA-MB-231-luc xenograft mice. The bottom tumor is targeted during gradient-localized heating, as described in the graph. (**C**) Luciferase bioluminescence activity of the tumor was measured before and after treatment. The results show a greater than six-fold decrease in activity for the treated bottom tumor, while the untreated tumor had almost no change in bioluminescence activity. Thus, this demonstrates the utility of MPI gradients in localizing tumor therapy. Adapted with permission from Ref. [364], Copyright 2018 American Chemical Society. (**D**,**E**): magnetic nanocapsules for multimodal-imaging and magnetically guided combination cancer therapy. (**D**) Schematic illustration of the gold-silica nanocapsules loaded with DOX and of the employed combination therapy. (**E**) (**a**,**c**) In vivo T_2_ -weighted MR images of 4T1 tumor-bearing mice at various time points postinjection of magnetic nanocapsules without (**a**) or with (**c**) magnetic tumor targeting. White circles indicate the positions of tumors. (**b**,**d**) In vivo PA images of the tumor sites at different time points, postinjection of the magnetic capsules without (**b**) or with (**d**) magnetic targeting. Reprinted with permission from Ref. [401], Copyright 2016 American Chemical Society. (**F**,**G**) MRI and MPI of 1  ×  10^6^ ferumoxytol-labeled MSCs implanted within the hind limb muscle of mice; imaging was performed 1 day (**F**) or 12 days (**G**) post implantation. Yellow and blue arrows indicate the transplantation site, while red triangles indicate misleading signal coming from the bone (in MRI) or from the iron in the gut of the mice (in MPI). (**H**) Prussian Blue staining with nuclear fast red counterstain confirms the presence of iron within MSCs. Adapted with permission from Ref. [409].

More recently, MFH was co-performed with real-time MPI [389]. A combined MPI–MFH scanner was developed, in which seamless switching between imaging and therapy while the subject remains in the scanner was possible. This system is capable of selectively heating SPIO particles separated by a distance of 7 mm, and providing imaging at a high spatial resolution. As a proof of concept in vivo, mice bearing two tumor xenografts of human origin each were injected with SPIONs, which were then visualized by MPI (Figure 11A–C) [364]. One only tumor in the couples was selectively heated by MFH, while the other tumor served as the negative control. Ex vivo studies proved that heat damage was indeed localized to the target tumor, while the off-target tumor or off-target healthy clearance organs were spared.

MCs for simultaneous MPI and MFH must have a high SAR and reach magnetization saturation at a small magnetic field. Even if IONPs are promising as MFH agents, they have not yet been translated to the clinical practice of this application due to the following reasons: (i) the low heating power of the clinically approved IONPs; (ii) their limited ability to target tumor cells; and (iii) their inhomogeneous distribution in the tumor tissue. The direct injection of magnetic nanoparticles into the tumor is necessary to achieve sufficient local concentrations of particles that make the MFH treatments effective [390].

#### 5.2.2. Imaging of Targeted Drug Delivery from Smart Systems

Magnetically targeted drug delivery has been already reviewed in paragraph three, however, the possibility to image this process has not been thoroughly treated yet. One of the great advantages of using MCs resides not only in the possibility of driving them from the external, but also in the possibility of tracking this process [391]. The basic requirement is the stability of the co-loading of the drug and the imaging agent. If the drug is released before the imaging agent, in fact, the information gained is not reliable. This does not apply to MCs employed for MFH, as the magnetic system is the therapeutic agent itself. Moreover, as mentioned in paragraph three, MCs used for drug delivery should be biocompatible and their clearance should be quite fast, in order to allow multiple drug delivery administrations in a selected period of time. Among the imaging techniques employed to follow drug delivery from MCs, MRI is the most exploited, however, also PAI, OI, and MPI can be employed. Svenskaya et al., for example, recently developed and optimized biodegradable capsules sensitive to magnet navigation, demonstrating the possibility of tracking their delivery by MRI in a breast cancer murine model. For this purpose, an external magnet was applied for one hour on the mouse tumor, then the MRI contrast was followed up 48 h post-injection [100]. Guo et al. endowed methotrexate thermosensitive magnetoliposomes with both the lipophilic fluorescent dye Cy5.5 and magnetic nanoparticles in the bilayer, and DOX in the core. By the combination of magnetic and receptor targeting, the administered nanosystem reached the tumor, and thanks to the application of an alternating magnetic field the drug can be selectively released. Drug delivery was tracked both by MRI and Optical Imaging (OI) [392]. Foy et al. demonstrated the possibility of combining magnetic targeting with OI to track drug delivery of a hydrophobic anticancer drug to breast cancer tumors, using magnetic nanoparticles [393,394]. Recently, MPI was also employed to track magnetic guided delivery, even if this field is still in its infancy. Zhang et al. proposed a real-time imaging-based guidance system for simultaneous monitoring and actuation of MCs. However, this system was limited to 1D monitoring [395,396]. Le et al. later developed a 2D monitoring system that rendered magnetic particle imaging-based navigation more feasible [397]. Meantime Yu et al. demonstrated for the first time the use of MPI for in vivo cancer imaging with systemic drug-free tracer administration [398], while Jung et al. reported the MPI tracking of SPIO-labeled therapeutic exosomes distribution in vivo in breast cancer-affected mice [399]. However, simultaneous magnetic guidance and drug targeting imaging by MPI has not been reported yet. Interestingly, while monitoring the intratumoral release of Doxorubicin from Fe_3_O_4_-PLGA core-shell nanocomposites, Zhu et al. found a strong correlation between the MPI signal and the % of doxorubicin release, and demonstrated that ∼67% of doxorubicin molecules in nanocomposites was released in the tumor site within the first 48 h after injection [400]. Concerning PAI of magnetically controlled drug delivery, only a few papers have been published [396]. Huang et al. designed a “yolk-shell” structure, confining movable gold nanorods in iron oxide nanoshells loaded with DOX and functionalized with hyaluronic acid. The obtained system was injected intravenously and magnetically driven to a 4T1 tumor. Drug delivery was successfully imaged by both PAI and MRI (Figure 11D,E) [401]. 

#### 5.2.3. Imaging of Cell Therapy In Situ

The use of cell therapies in clinical trials is constantly increasing [402]. The success of these therapies depends entirely on the accurate delivery of cells to the target organs. Therefore, having the ability of labeling cells and tracking them directly in vivo with high spatial resolution and high sensitivity is of utmost importance to predict the therapy outcome. The main cell therapies adopted employ two different kinds of cells: immune and stem cells [403]. The most diffuse and sensitive method to track them makes use of magnetic systems [346]. It consists of labeling them with SPIO and monitoring them by MRI after the administration [346,404,405]. The advantages of using MRI are represented by the high spatial resolution, the co-registration of anatomical images that allow to spatially locate administered cells, the excellent soft tissues contrast, and the absence of ionizing radiations [406]. The first effective MRI tracking of ex vivo MCs labeled cells in human patients was reported in 2005 by I. de Vries et al. [403]. In more detail, they labeled autologous dendritic cells with SPIO and tracked them after ultrasound-guided intranodal administration in melanoma patients. As a result, MRI allowed the assessment of the accuracy of dendritic cell delivery and inter- and intra-nodal cell migration patterns. They estimated that 1.5 × 10^5^ migrated cells and ∼2 × 10^3^ cells/voxel could be visualized. In this case, the immature dendritic cells naturally endocytosed SPIO, but in the case of non-phagocytic cells, the addition of transfection agents is generally required [407]. Of course, both the imaging and the transfection agents should not affect the cell function, viability, phenotype, and receptor expression. The advantage of labeling cells with MCs resides also in the possibility of guiding them to the pathological site selectively using pulsed magnetic field gradients or applying a local external magnet [269,281,295]. Besides the possibility of controlling cells remotely, moreover, labeling cells with MCs allows for cell function modulation, by external magnetic fields application [131,137,205]. This chance is of the utmost importance, especially in the field of cell therapy for tissue regeneration, as already reviewed in Section 3.2. 

Even if MRI is undoubtedly the main imaging technique employed to track MCs labeled cells, MPI, PAI, and US have also been recently proposed. MPI cell tracking displays great potential for overcoming the challenges of MRI-based cell tracking allowing for both high cellular sensitivity and high specificity and quantification of SPIO labeled cell numbers, even if the technique still needs to be fine-tuned (Figure 11F–H) [408,409]. Dhada et al. developed ROS-sensitive gold nanorods for mesenchymal stem cells tracking by PAI, with the possibility of discerning between viable and dead stem cells with high spatiotemporal resolution. They found that, after transplantation of mesenchymal stem cells into the lower limb of a mouse, a significant cell death occurred within 24 h, with an estimated 5% viability after 10 days [410]. Donnelly et al. used gold nanospheres to label MSCs and track them during and after the injection in the spinal cord by both PAI and US, detecting as few as 1 × 10^3^ cells [411]. CT can also be employed to track MCs labeled cells [412], but with a great limit on the use of ionizing radiations, which hampers assiduous monitoring. Of course in the present paragraph, only some of most representative studies about cell tracking, combined with MCs cell labeling, were reported, just to give to the reader the idea of the great significance of this topic for future medical applications.

## 6. Perspectives

Targeting bioagents via magnetic forces has several key advantages. First, magnetic targeting uses a strong targeting power with high penetration depth, which can facilitate the administration of medications in the human body [153]. Second, it is safe and compliant with use in living systems, as the side effects of MCs are limited. Third, it has the potential for high therapeutic selectivity. The therapeutic selectivity refers not only to the ability to reach targets at a high molecular resolution (e.g., recognition of specific disease-associated molecules) and with patient-specificity (e.g., targeting of molecular fingerprints of tumors) but also to the opportunity to establish mechanisms for conditional and controlled release of the bioagents within the pathological site. As such, not only MCs are more promising for drug delivery as compared to other vehicles and vehiculation mechanisms, but they also are helpful in other in vivo applications, such as biosensing. For instance, MCs can serve as bioprobes to image malignancies and diseases, or to detect biomolecular interactions [158,384,396]. 

In the future, MCs will continue to gain medical relevance as applied to various uses, including theranostics, separation of biomolecules, sorting of diseased cells, manipulation of genetic material, and control of bioagents in vivo. Nevertheless, to render MCs successful delivery platforms, several requirements need to be addressed [121]. One first important assumption is that MCs are developed to possess sufficient magnetization making them responsive to the application of external magnetic fields. This property of MCs largely depends on their size, which in turn affects the applicability within living systems [157]. Another important feature in MC-based pharmaceutical vehicles is the long-circulation, which can be realized by implementing stealthy systems that avoid the clearance operated by the phagocyte system [184,195]. Magnetic controllability and stability in vivo are vital prerequisites for the future development of MCs as multifunctional platforms. In this regard, the most promising directions for next generation MCs are effective mechanisms to control the drug uptake and release, as well as designs that permit theranostic functions [157]. An essential aspect that still requires intense development is the interaction of the MCs with living environments. Ideal MCs should be able to efficiently move within the bodies of living systems. Depending on the application and the used MCs type, guiding magnetic materials within the body requires one to overcome several issues [325,413,414,415]. Among them, the anatomical structures represent a major physical barrier that hinders controlled movement at different levels (from micro to macro scale). The physical properties of injectable formulations of MCs must be compliant with the purpose of navigation within the vascular networks. As an example, not only the size and morphology of the MCs must be optimized for safe circulation within vessels, but also the systems’ stability has to be maximized to reach both long circulation time and reduced risk of releasing unsafe degradation products into the circulation. Stealthy designs can dramatically extend the circulation time while masking the carriers from clearance cell effectors and protecting them from excretion [416,417]. At the same time, successful intravascular guidance of the MCs must win the forces generated by the hemodynamics [418]. To guide MCs through compact tissues, the magnetic attraction must overcome the physical barriers of the matrix and other tissue components. In addition, as cells are responsive to chemotactic cues (such as the chemokine gradients generated by other cells within pathological sites), fully controllable dragging of magnetized cells should also overcome the natural chemophoretic tendency that cells experience in their specific microenvironment [419,420]. As far as we can argue from the literature, the trend in research points out the sophistication of MCs for the targeted delivery of bioagents [157]. MCs are in fact increasingly designed to become multifunctional, while at the same time being performant in relation to their basic abilities as carriers that, as mentioned above, mostly refer to their in vivo structural stability and controllability of the bioagent liberation [157,169,202].

Even if tremendous progress was recently achieved in the MCs’ development, the actual applicability of these systems needs to be discussed. MNPs have received FDA approval and have been commercialized for use as MRI contrast agents and hyperthermia [211,380]. Nevertheless, their use in magnetic targeting is not yet at a stage of clinical relevance. Some limited clinical trials (even in Phase III) have already started, but none of them has yet resulted in FDA approval or commercialization [421,422]. One example is a recent study from Kamei et al., which concluded that magnetic targeting of ferucarbotran-labeled MSCs could be performed safely in five patients with cartilage defects in the knee joint [301]. A compact magnet (one T) was placed around the knee joint of patients to allow the magnetic force to be as perpendicular to the surface of the lesion as possible. The magnet was maintained in position for 10 min after the injection of 1 × 10^7^ magnetized cells into the knee joint. Upon magnetic targeting, the injured sites were completely refilled with cartilage-like tissues and the patients displayed improved clinical outcomes 48 weeks after treatment. Hence, magnetic cell guidance might represent a minimally invasive treatment for cartilage repair.

Magnetic cell guidance is a peculiar instance of magnetic targeting. In the last years, magnetic materials have attracted much attention for other applications in addition to specific drug release [423]. For instance, an interesting use of magnetic actuation is to control the process of the self-assembly of multicellular aggregates. Cells, cells spheroids, or matrices containing cells can be modified with magnetic particles and then, under the application of a magnetic field, can be induced to assemble into morphologies that mimic the structure of target organs (e.g., rings for vessels, sheets for skin, etc.) [110,424]. Thus, magnetic tissue engineering emerges as an important tool to enhance bioprinting functionality and realize future multiplex bioconstructs and organ replicas. Moreover, MCs are also promising for robotics as applied to drug delivery and regenerative medicine [305,323,324,327]. Thus far, microrobotic MCs have been widely studied in vitro and scarcely in animal models, with no reported use in humans [305,425,426,427,428]. Nevertheless, more and more reports validate the performance of their robot in phantoms that replicate the anatomical structures of patients as realized from imaging data [426,429]. Although this field is moving forward in the direction of clinical applicability, consistent efforts are still needed before starting experimentation in humans [426].

One of the major problems is to magnetically control the delivery of bioagents in-depth in the body as realized with the practical implementation of the magnetic fields. In-body sources of magnetic fields (like magnetized or magnetic scaffolds) have been proposed as a solution for portability [279,425,430], which might enhance the targeting efficiency when vehiculating drugs or guiding the homing of medical cells. However, the use of magnetic implants requires surgical intervention and it is not always possible. Hence, more research effort is needed to expand the applicability spectrum of magnetic targeting, as well as its implementation in real-life use.

## Figures and Tables

**Table 1:** In vivo studies of magnetic cell targeting categorized in relation to the target organ.

Target Organ	Injected/Transplanted Cells	Magnetizing Material	Recipient	Animal Model	Cell Delivery Modality	Magnetic Set Up	Magnet Position	Cell Accumulation (Increase Folds)	Time Point	Ref.
**Heart**	Cardiosphere-derived cells (rat)	Superparamagnetic microspheres (diameter: 0.9 μm, Bangs Laboratories)	Rat	Cardiac ischemia	Intramyocardial injections	Magnet (1.3 T) was applied during the injection and the following 10 min	External, above the apex	2–3	24 h	[265]
	Cord blood EPCs (human)	Anionic citrate-coated maghemite nanoparticles (diameter: 8 nm)	Rat		Cardiac intraventricular injection	Neodymium magnet (0.1 T, magnetic gradient 11 T/m at 4 mm from its surface) applied for 24 h	Subcutaneously implanted	10	24 h	[274]
	Cardiosphere-derived cells (rat)	Superparamagnetic microspheres	Rat	Myocardial infarction	Cardiac intraventricular injection	Circular NdFeB magnet (1.3 T)	External	4 3	24 h 3 weeks	[264]
	MSCs (rat)	Ferucarbotran/Resovist	Rat		Intracoronary infusion	Permanent Neodymium-iron-boron (NdFeB) magnet cylinder	External	3.9		[275]
	MSCs (rat)	Ferucarbotran/Resovist^®^ (diameter: 62 nm)	Rat		Transjugular injection into the left cardiac vein	Permanent NdFeB magnet cylinder (0.6 T; diameter: 8 mm)	External	2.7–2.9	24 h	[276]
	Cardiosphere-derived stem cells (human)	Ferumoxytol (Feraheme^®^)	Rat	Myocardial infarction	Intracoronary injection	A permanent NdFeB magnet (1.3 T) was applied during the injection and the following 10 min	External	3	24 h	[272]
	MSCs (pig)	Gadolinium nanotubes and Molday ION(–)^®^	Pig	Cardiomyoplasty	Transepicardial injection	Permanent a 1.3 T NdFeB ring magnet	Implanted (sutured onto the cardiac ventricle)	3	24 h	[277]
	MSCs (rat)	Ferucarbotran/Resovist^®^ (diameter: 62 nm)	Rat		Cardiac intraventricular injection	Cylindrical NdFeB magnets (0.15 T, 0.3 T, and 0.6 T)	External	4	24 h	[278]
**Vascular system**	Endothelial progenitor cells (pig)	Superparamagnetic microspheres (diameter: 0.9 μm, Bangs Laboratories)	Pig		Infusion in the central lumen of an angioplastic balloon	Magnetized stent	Implanted	6–30	24 h	[263]
	Aortic endothelial cells (bovine)	Polylactide MNPs (diameter: 290 nm)	Rat		Transthoracical cardiac intraventricular injection	Magnetic field source of 500 G	Implanted (magnetized stent)	6	2 days	[279]
	Endothelial cells (human) (HUVECs)	MNPs-TransMAG, Chemicell	Mouse	Denuded carotid artery	Injection in external carotid artery	3 small NdFeB magnets (diameter: 1 mm; 10-mm length)	External	Improved (nonquantitative)	24 h	[280]
	Endothelial progenitor cells	SPIO Endorem	Rat		Infusion in the common carotid artery	Magnetic array	External	5.4	24 h	[281]
	MSCs (rabbit)	FluidMAG-D^®^ (Chemicell)	Rabbit		Infusion in the central lumen of the over the wire angioplastic balloon	Permanent cylindrical magnet (Halbach cylinder)	External	6.2	24 h	[268]
**Brain**	Neural stem cells (human)	Ferumoxide	Rat	Ischemia	Intravenous injection (tail vein)	Neodymium magnet	External (attached to the skull for 7 days)	6	7 days	[282]
	Endothelial progenitor cells (mouse)	SPIONs (diameter: 50 nm)	Mouse		Intravenous injection (tail vein)	Two permanent FeNdB magnets (3 × 42 mm; 0.3 T)	Implanted (left hemisphere)	Non defined	24 h	[283]
	Embryonic stem cell (ESC)-derived spherical neural masses (human)	FIONs (60 nm)	Rat	Intracerebral hemorrhage	Intravenous injection (tail vein)	Neodymium magnet (5 × 10 × 2 mm^3^; 0.32 T)	External (helmet)	2	3 days	[284]
	Human nasal turbinate stem cells	SPIONs (diameter: 15–20 nm)	Mouse		Intranasal delivery	Permanent cylindrical magnet	External (attached to the restrainer)	Non quantitative	2 days	[285]
**Knee joint (cartilage)**	MSCs (rat, human)	Feridex (Tanabe Seiyaku)	Rabbit, pig	Osteochondral defect	Local transplantation (knee joint)	Permanent	External	Improved (nonquantitative)		[250,286]
	MSCs (rabbit)	Ferucarbotran/Resovist^®^	Rabbit		Local transplantation	Permanent	External			[287]
**Spinal cord**	MSCs (rat)	Poly-L-lysine-coated SPIONs	Rat	Osteochondral defect	Intrathecal transplantation	Permanent; Neodymium magnet (13 × 7 × 2 mm; 0.35 T; remnant magnetic field B_r_ = 1.2 T)	Implanted at 4–4.5 mm above the lesion site	3.7	1 week	[288]
	MSCs (rat)		Rat	Contusion-derived injury	Subarachnoidal injection	Permanent; Neodymium magnet	Implanted in paravertebral muscles, T7 level)	30	24 h	[289,290]
	MSCs	SPIONs	Rat	Lesion	Intrathecal transplantation (L5–L6 level, 10 cm from the lesion)	Permanent; two cylindrical NdFeB magnets (1 cm × 5 cm; remanent magnetization: 1.2 T)	External	4	2 days	[291]
**Lung**	MSCs (human)	DMSA-coated maghemite nanoparticles	Mouse	Silicosis	Systemic inoculation	Permanent; Neodymium circular magnets (20 mm × 2 mm)	External, onto the thoracic region	1.5	2 days	[292]
**Skeletal muscle**	MSCs (human)	Ferucarbotran/Resovist^®^	Rat	Local damage	Local transplantation (between the fascia and scar tissue)	Electromagnet (magnetic field intensities from 0 to 5 T)	External	3–20	2 days	[293]
**Bone**	MSCs (rat)	Ferucarbotran/Resovist^®^	Rat	Non-healing fracture	Percutaneous injection	Permanent superconducting bulk magnet system (3T)	External	2–3	3 days	[271]
**Retina**	MSCs (rat)	FluidMAG-D^®^ (Chemicell)	Rat	Retinal degeneration (S334ter-4 transgenic rats)	Intravitreal or tail vein injection	Gold-plated neodymium disc magnet	Implanted within the orbit, but outside the eye	37 (intravitreal injection) 10 (intravenous injection)	1 week	[294]
**Tumor**	Macrophages (human)	SPIONs (25 nm)	Mouse	Prostate tumor (in dorsolateral prostate) Lung tumor metastasis	Intravenous injection (tail vein)	MRI Scanner: small bore magnet (7 T, gradient of 660 mT m^−1^)	External	Improved (non quantitative)	1 h	[295]
	CD8^+^ T cells (mouse)	Magnetic nanoparticles (80 nm)	Mouse	Lymphoma (from E.G7-OVA cell line), subcutaneous in the flank	Intravenous injection (tail vein)	Permanent; neodymium magnet (8 × 6 mm)	External magnet, applied over the tumor	Improved (non quantitative)	2 weeks	[296]
	CD8^+^ T cells (mouse)		Mouse	Lymphoma (from E.G7-OVA cell line) Breast cancer (from 4T1 cell line)	Intratumoral injection					[297]
	NK-92 cell (human)	CD56-conjugated magnetic nanoparticles (75 nm)	Mouse	Hepatoma (from H22 cell line) Melanoma (from B16 cell line)	Intravenous injection (tail vein)	Permanent magnet	External magnet, applied over the tumor (6h)	Undefined (non quantitative)	2 weeks	[298]
